# Pick-up single-cell proteomic analysis for quantifying up to 3000 proteins in a Mammalian cell

**DOI:** 10.1038/s41467-024-45659-4

**Published:** 2024-02-10

**Authors:** Yu Wang, Zhi-Ying Guan, Shao-Wen Shi, Yi-Rong Jiang, Jie Zhang, Yi Yang, Qiong Wu, Jie Wu, Jian-Bo Chen, Wei-Xin Ying, Qin-Qin Xu, Qian-Xi Fan, Hui-Feng Wang, Li Zhou, Ling Wang, Jin Fang, Jian-Zhang Pan, Qun Fang

**Affiliations:** 1 Institute of Microanalytical Systems, Department of Chemistry, Zhejiang University, Hangzhou, 310058 China; 2 Single-cell Proteomics Research Center, ZJU-Hangzhou Global Scientific and Technological Innovation Center, Hangzhou, 311200 China; 3 College of Computer Science and Technology, Zhejiang University, Hangzhou, 310027 China; 4 Department of Cell Biology, China Medical University, Shenyang, 110122 China; 5 Shanghai Omicsolution Co., Shanghai, 201100 China; 6 Key Laboratory of Excited-State Materials of Zhejiang Province, Zhejiang University, Hangzhou, 310007 China

**Keywords:** Proteomic analysis, Microfluidics, Proteomics

## Abstract

The shotgun proteomic analysis is currently the most promising single-cell protein sequencing technology, however its identification level of ~1000 proteins per cell is still insufficient for practical applications. Here, we develop a pick-up single-cell proteomic analysis (PiSPA) workflow to achieve a deep identification capable of quantifying up to 3000 protein groups in a mammalian cell using the label-free quantitative method. The PiSPA workflow is specially established for single-cell samples mainly based on a nanoliter-scale microfluidic liquid handling robot, capable of achieving single-cell capture, pretreatment and injection under the pick-up operation strategy. Using this customized workflow with remarkable improvement in protein identification, 2449–3500, 2278–3257 and 1621–2904 protein groups are quantified in single A549 cells (*n* = 37), HeLa cells (*n* = 44) and U2OS cells (*n* = 27) under the DIA (MBR) mode, respectively. Benefiting from the flexible cell picking-up ability, we study HeLa cell migration at the single cell proteome level, demonstrating the potential in practical biological research from single-cell insight.

## Abstract (web summary)

Single-cell proteomics is of fundamental importance to capture biological heterogeneity, while limited in proteome depth. Here, the authors develop a pick-up single-cell proteomic analysis (PiSPA) workflow to achieve a deep coverage of quantifying up to 3000 protein groups in a mammalian cell.

## Introduction

Nowadays, single-cell genomic^1^ and transcriptomic technologies^2^ have been well developed. However, the development of the single-cell proteomic technology faces major technical challenges, because the protein content in a single cell is extremely low and proteins are difficult to be amplified as nucleic acids. So far, a variety of protein analysis techniques at the single-cell level have been developed, including those based on specific antibody labeling such as flow cytometry^3^, fluorescence imaging^4^, Western blotting^5^ and mass cytometry^6^, as well as those based on non-labeling proteome analysis technique with high-resolution mass spectrometry (MS). At present, among the proteomic analysis techniques based on MS, the shotgun technique using the bottom-up strategy usually demonstrates the maximum depth and breadth of protein identification^7^, with which usually 5000–7000 proteins^8–10^ can be identified in a single measurement with a liquid chromatography-mass spectrometry (LC-MS) system for a sample containing a large number of cells with a protein amount in the range of 200–1000 ng. Therefore, in recent years, a variety of single-cell proteome analysis approaches based on the shotgun technique have been developed^11–24^.

A typical single-cell shotgun proteomic analysis process includes the sorting of target cells from a large number of sample cells, the pretreatment of single-cell samples, the LC injection and separation, and ESI-MS/MS detection of the digested peptides. The sample pretreatment process includes multi-steps of cell lysis, protein reduction, alkylation, enzymatic digestion and termination of the digestion. For such single-cell samples with extremely small protein amounts (ca. 100–500 pg per cell^25^), if the above-mentioned series of sample pretreatment operations are carried out with a microliter-scale reactor such as a centrifuge tube^11^ or a multi-well plate^26^, obvious sample loss will occur during the sample pretreatment and transferring process, which will significantly reduce the number of protein identification and thus severely limit the identification depth of single-cell proteomics.

To break through the barriers of the identification depth of single-cell proteomics, one strategy to address the above challenge is to perform the sample pretreatment in nanoliter-scale in-situ microreactors, such as the oil-air-droplet (OAD) chip^13^, the integrated proteome analysis device (iPAD)^14^ and the nanodroplet sample preparation (nanoPOTS) platform^12,16,27–30^. Compared with the conventional microliter-scale reactors used in routine laboratories, these microreactors have a volume reduction of hundreds of times, which can avoid the excessive dilution of the trace amounts of single-cell samples, effectively improve the reaction efficiency and reduce the sample loss caused by the adsorption of the sample components on the reactor surface during the pretreatment process. Based on these methods, up to 300–1100 proteins were able to be identified from single cells with label-free approach^12,14,27,29^. With the tandem mass tag (TMT) approach, the maximum up to 1500 proteins were quantified from single acute myeloid leukemia (AML) cells^31^. However, most of the reported nanoliter-scale microreactors required to use microfabricated microchips or devices as the microreactors, and needed special sample injection devices and additional operations to complete the injections of the nanoliter-volume samples. Currently, an important emerging trend is to develop single-cell proteomic platforms capable of integrating whole-process operations for promoting the practicality and popularization of single-cell proteomic analysis. Some integrated platforms were developed by using the combination of commercial instruments and self-developed systems (such as the autoPOTS platform^32^, T-SCP platform^19^, SCeptre^33^ and One-Pot^34^ workflow) or an integrated microfluidic chip^17,18,35,36^, to complete the whole process of single-cell proteomic analysis, with 300–2000 proteins identified from single cells.

In spite of the significant progresses obtained in single-cell proteomics, how to further improve the protein identification depth and simultaneously simplify the device and operation to achieve practical whole-process proteomic analysis at the single-cell level still presents great challenges.

**Fig. 1:**
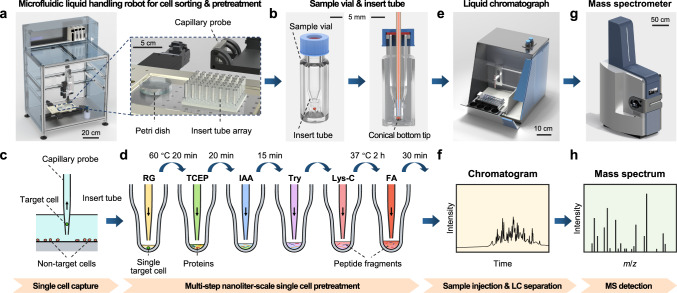
Schematic diagram of the PiSPA workflow for single cell proteomic analysis. The PiSPA workflow was conducted using a probe-based microfluidic liquid handling robot for cell sorting and pretreatment, a commercial LC system with an autosampler, and a tims-QTOF mass spectrometer. The microfluidic liquid handling robot (**a**) with an insert tube array (**b**) completed the sorting of single cells and the multi-step pretreatment of the single cell samples with the automated pick-up operation mode, including sorting of single target cells (**c**), nanoliter-scale cell lysis (RG, RapiGest SF), protein reduction (TCEP, tris (2-carboxyethyl) phosphine), alkylation (IAA, iodoacetamide), enzymatic digestion (Try, trypsin; Lys-C, Endoproteinase Lys-C) and termination of the digestion (FA, formic acid) (**d**). Insert tubes coupled with sample vials were used as the nanoliter microreactors for sample pretreatment of single cells (**b**). After sample pretreatment, the insert tubes & sample vials were used as sample tubes for the autosampler of the LC system to perform the sample injection (**e**), LC separation (**f**) and subsequent MS detection of the digested peptide components from single cells (**g**, **h**).

Here, we develop a total workflow solution for single-cell proteomic analysis (Fig. 1) capable of achieving deep identification quantifying up to 3000 protein groups in a single mammalian cell. More automatic and convenient operation is performed by using a probe-based microfluidic liquid handling robot coupled with a commercial liquid chromatograph (LC) and a trapped ion mobility spectrometry (TIMS) QTOF mass spectrometer. The automated pick-up operation mode based on capillary probes is adopted throughout the pick-up single-cell proteomic analysis (PiSPA) workflow, including the sorting of single cells and multi-step single-cell pretreatment to digest cellular proteins into peptides in nanoliter reactors, as well as the injection of the peptide samples to the LC column. In addition, we utilize a single-cell customized strategy that fully considered the effects of the unique properties of single cells *vs*. bulk cells on sample pretreatment, separation, and detection to establish the series of measures throughout the PiSPA workflow. This strategy enables a much deeper depth of protein identification in single-cell analysis than those previously reported in the literatures. We apply this platform in the single-cell proteomic analysis of three kinds of mammalian cells, HeLa, A549 and U2OS cells, as well as the single-cell proteomic study of migrating HeLa cells.

## Results

### Establishment of the PiSPA workflow

We first integrated an improved microfluidic liquid handling robot^37^ previously developed by the authors’ group based on the sequential operation droplet array (SODA) technique^38,39^ into the PiSPA platform to achieve nanoliter-scale single-cell sorting and multistep pretreatment under the probe pick-up mode. The PiSPA platform performed the cell sorting with three-step operations of single-cell identification, picking-up and dispensing. Under the pick-up mode, it achieved the automated bright-field or fluorescence imaging and identification of the target cells in the cell suspension samples based on their bright-field apparent property or labeled fluorescence signals, picking-up of the single target cell by a tapered capillary probe connected with a high-precision syringe pump, and dispensing the target cell into an insert tube. We evaluated the cell sorting performance of the PiSPA platform by using it to pick-up 20 single HeLa cells separately and dispensing them individually into different insert tubes. Under the optimized conditions (i.e. capillary tip diameter of 35 μm, aspirating flow rate of 500 nL/s, aspirating volume of 25 nL, and injection volume of 400 nL), at least 19 single HeLa cells were successfully dispensed into the insert tubes, corresponding to a single-cell assignment rate of 95% (*n* = 20). To ensure the accuracy of cellular protein analysis, we used freshly isolated or cultured cell samples. The entire procedure of identification, picking-up and dispensing for each target cell usually took 20 s. Therefore, sorting of dozens of single cells could be achieved in 10–20 min. We observed that after the picked HeLa cells were dispensed into PBS droplets, the morphology of these cells was intact and they could be attached to the bottom surface of the vessel. A fluorescent dye for detecting cellular activity were also used to stain the cells, showing that they could retain their viability. Typical images of a picked HeLa cell in a droplet before and after staining by the fluorescent dye for cell viability assay are shown in Supplementary Fig. 1.

After the probe captured the target cell, we controlled the platform to dispense the cell into a commercial insert tube instead of using a microchip as the microreactor as previously reported^13^. The insert tubes can be seamless compatible with the autosampler of commercial LC-MS systems, without the need for microfabricated microchips and troublesome sample injection operations^13,26,29^. However, even for the smallest insert tubes available on the market, their volumes are still hundreds of microliters, which are far from a nanoliter-scale microreactor required for single-cell sample pretreatment. We solved this difficulty using the conical bottom tip (2 mm inner diameter, 0.4 mm height) of the insert tubes to load the nanoliter reaction solution. In addition, the evident evaporation effect of the nanoliter reaction solutions during the heating reaction process was suppressed by adding 100 μL of water and a sealing cap to the sample vial outside the insert tube to form an internal high-humidity environment. With the precise manipulation ability for nanoliter-scale liquids, the single-cell capture platform was continuously adopted to complete the multi-step nanoliter-scale reagent addition operation to sequentially achieve cell lysis, protein reduction, alkylation, digestion and reaction termination, under the pick-up operation mode. We performed a comparison experiment to conduct sample pretreatment of single HeLa cells with reaction volumes of microliters (5 μL) and nanoliters (~400 nL). The protein identification number of the nanoliter reactors was double that of the microliter reactors (Supplementary Fig. 2). The advantage of nanoliter-scale reactors in single cell proteomic analysis had also been demonstrated in many previous literatures^14,25,30^.

**Fig. 2:**
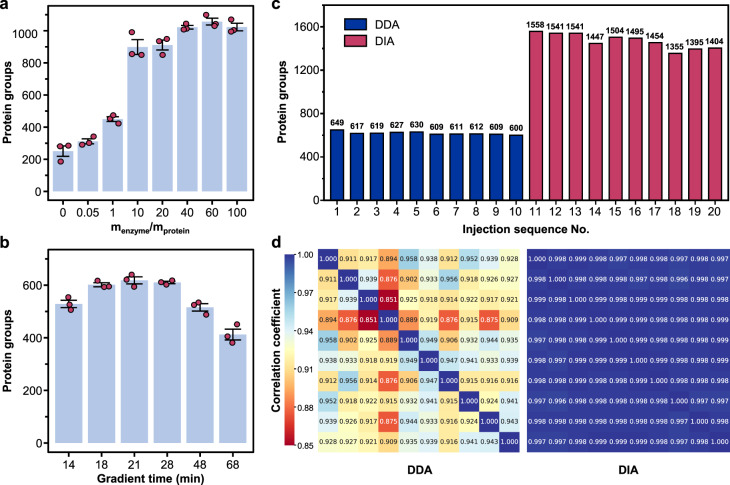
System optimization and performance. **a** Enzyme/protein ratio optimization for single-cell proteomic analysis. Under the DDA mode, an average of 251, 312, 450, 899, 911, 1022, 1057, and 1023 protein groups (*n* = 3) were quantified at m_enzyme_/m_protein_ of 0, 0.05, 1, 10, 20, 40, 60, and 100, respectively. Conditions: Sample, single HeLa cells; LC gradient time, 21 min. **b** Optimization of LC gradient time. Under the DDA mode, an average of 529, 602, 618, 610, 516, and 412 protein groups (*n* = 3) were quantified with LC gradients of 14, 18, 21, 28, 48, and 68 min, respectively. Conditions: Sample, 200 pg of standard HeLa cell digestion; enzyme/protein ratio, 50:1. In (**a**, **b**), the bars indicate the mean values of the corresponding data; the error bars indicate the standard deviations; individual data points are overlaid. **c**, **d** Test for the repeatability of 10 consecutive analysis of 200 pg standard HeLa cell digestion sample using the LC-MS system in DIA and DDA modes, with a LC gradient time of 21 min. The results of protein identification number under the DIA and DDA mode (**c**) and their Pearson correlation coefficients of pairwise analysis (**d**) are shown. Source data are provided as a Source Data file.

In the protein digestion experiments, we studied the effect of the enzyme/protein ratio on the protein identification number using single HeLa cells as samples. In routine proteomic experiments, enzyme/protein ratios between 1:100 and 1:10 are usually adopted as the optimized digestion conditions. Most of the current single-cell proteomic workflows also followed this enzyme/protein ratio range^13,16,18,19,30,31^. However, our experimental results (Fig. 2a) showed that the single-cell protein identification number showed an increasing trend with the increase of the enzyme/protein ratio. This increasing trend showed a rapid change in the enzyme/protein ratio range of 0-15:1, and significantly slowed down after 15:1. When the enzyme/protein ratio was between 40:1 and 60:1, the protein identification number reached the highest level (~1100 protein groups) and the fluctuations (i.e. CV) in the protein numbers identified in different single cells were also at the lowest level. Comprehensively considering the protein identification number and the working stability of the platform, we finally chose an enzyme/protein ratio of 50:1 in the subsequent single-cell proteomic analysis experiments. Such an enzyme/protein ratio largely exceeding those used in most conventional and single-cell proteomic experiments, was favorable to maintain a relatively high enzyme concentration^40,41^ (e.g. ~12 μg/mL trypsin in the present platform) in the nanoliter-scale reaction solutions to increase digestion efficiency of proteins. Another possible benefit of using larger amounts of enzymes may be related with the scale characteristics of single-cell analysis, that is, the largely excessive enzyme molecules in the reaction solutions could serve as an adsorption substitute, which substantially reduced the adsorption loss of trace cellular proteins and digested peptides on the surface of the microreactor and transmission conduit during the sample pretreatment and injection process.

To test the possible negative effects caused by the large enzyme/protein ratios, we first performed proteomic analysis for a control blank sample (containing all pretreatment reagents except for the single-cell sample), and no proteins from the two heterologous enzymes (trypsin and Lys-C) were identified and the protein identification number for the control samples was also at a very low level, indicating that the enzymes and their self-cleaved peptides did not produce misidentification of proteins. We also examined the peak positions and intensity signals of the self-cleaved peptides of trypsin in a proteomic analysis chromatogram of a typical single HeLa cell (Supplementary Fig. 3). It can be observed that the signals of the self-cleaved peptides of these enzymes appeared at only a few specific retention time and *m/z* positions and had no evident effect on the identification of the sample peptides.

The choice of gradient procedure in LC separations has a critical impact on chromatographic separation performance and proteome identification results. We evaluated the influence of different gradient times on single-cell proteomics analysis using gradients of 14, 18, 21, 28, 48, and 68 min with a sample of the 200 pg of standard HeLa cell digestion (Fig. 2b). The identification number reached the highest level of ca. 600 when the 18–28 min gradients were used, and then slowly decreased as the gradient time increased. Differing from the gradients of tens of minutes for routine proteomic analysis, the results showed that relatively short gradients are more beneficial for obtaining high protein identification numbers for single-cell samples. This is because short gradients are favorable for increasing the signal peak intensities of peptides, thereby improving the identification of some low-abundance peptide components in the sample (Supplementary Fig. 4). Another benefit of using short gradients is that it can increase the throughput of proteomic analysis. However, an excessively short gradient (such as 14 min gradient) will worsen the separation performance and result in a decrease in the number of protein identification. Comprehensively considering the analysis throughput and identification stability, we finally chose the 21-min LC gradient time in the subsequent experiments.

Before performing single-cell proteomic analysis of mammalian cells, we first tested the repeatability of the LC-MS system under the optimized conditions by running 10 consecutive analysis of the 200 pg of commercial HeLa cell digest serving as a quality control (QC) sample in data independent acquisition (DIA) and data dependent acquisition (DDA) modes. DDA and DIA are two widely-used MS data acquisition modes in proteomics analysis. DDA mode chooses a few most intense precursor ions based on the MS1 spectrum for acquisition of MS2 spectra and identification of peptides. In contrast, DIA mode allows the fragmentation of all precursor ions within a certain range of *m/z* and retention time to acquire complete record of theoretically all peptides in a sample^42,43^. On average, 1469 and 618 protein groups (Fig. 2c) with number variation coefficients of 4.7% and 2.3% were stably quantified under the DIA and DDA mode, respectively. The pairwise correlation analysis (Python package Pandas, version 1.4.4) between the protein quantification data of the 10 QC samples showed that all of the Pearson correlation coefficients were over 0.99 (DIA mode) and 0.85 (DDA mode) (Fig. 2d), demonstrating the good stability (especially in DIA mode) of the system in the proteomic analysis at the single-cell level.

### Single-cell proteomic analysis of mammalian cells

**Fig. 3:**
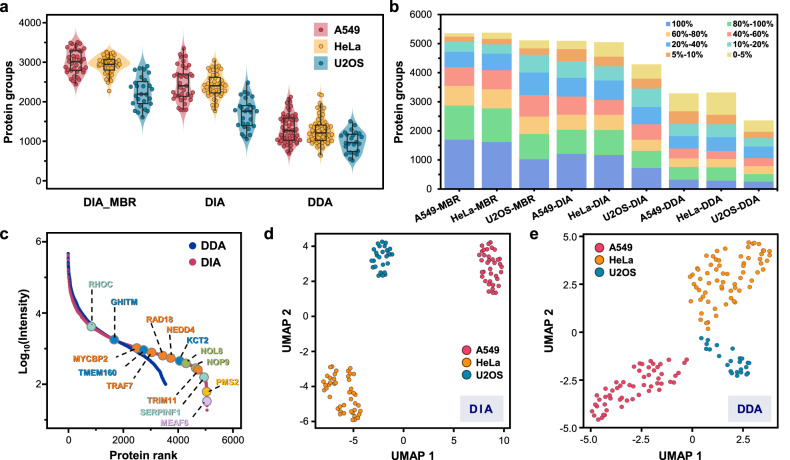
Application of the PiSPA platform in proteomic analysis of single mammalian cells. **a** Quantified protein group numbers of three types of mammalian cells (A549, HeLa, and U2OS cells) under the DIA and DDA modes. Under the DIA mode, an average of 3008 (2449–3500, median = 3013, *n* = 37), 2926 (2278–3257, median = 2946, *n* = 44) and 2259 (1621–2904, median = 2200, *n* = 27) protein groups were quantified in single A549, HeLa and U2OS cells with match-between-run (MBR) algorithm, and average of 2467 (1804–3349, median = 2409, *n* = 37), 2421 (1778–3049, median = 2409, *n* = 44) and 1705 (1074- 2487, median = 1770, *n* = 27) protein groups were quantified in the same single A549, HeLa and U2OS cells without MBR. Under the DDA mode, an average of 1328 (712–2129, median = 1282, *n* = 56), 1290 (664–2198, median = 1228, *n* = 68) and 1005 (536–1519, median = 984, *n* = 24) protein groups were quantified in single A549, HeLa and U2OS cells, respectively. Conditions: enzyme/protein ratio, 50:1; LC gradient time, 21 min. The central lines in the boxes indicate the median values of the corresponding data; the boxes indicate the quartiles; the whiskers extend to a maximum of 1.5 times interquartile range beyond the quartiles; individual data points are overlaid. **b** Recurrence percentage distributions of the protein groups quantified in single A549, HeLa, and U2OS cells in (**a**) under the DIA-MBR, DIA, and DDA modes. The bars indicate the mean values of the corresponding data; the error bars indicate the standard deviations; individual data points are overlaid. **c**, Rank order of protein abundance in single HeLa cells under the DIA (red dots) and DDA (blue dots) modes. Some of the proteins only quantified in the DIA mode are marked with colored points. **d**, **e** Comparison of UMAP dimensionality reduction analysis on the measured A549, HeLa, and U2OS cells in (**a**) under the DIA (**d**) and DDA modes (**e**). Source data are provided as a Source Data file.

Using the present workflow and platform, we performed single-cell proteomic analysis of three types of tumor cell lines, A549, HeLa, and U2OS cells, using the DIA and DDA modes, with the optimal enzyme/protein ratio of 50:1 and gradient time of 21 min. Under the DIA mode, an average of 2467 (1804–3349), 2421 (1778–3049) and 1705 (1074–2487) protein groups were quantified in single A549 cells (*n* = 37), HeLa cells (*n* = 44) and U2OS cells (*n* = 27), respectively (Fig. 3a). Using the match between runs (MBR) algorithm, an average of 3008 (2449–3500), 2926 (2278–3257) and 2259 (1621–2904) protein groups were quantified in the same samples of single A549, HeLa and U2OS cells, respectively. Under the DDA mode at the same analysis conditions, an average of 1328 (712–2129), 1290 (664–2198) and 1005 (536–1519) protein groups were quantified in single A549 cells (*n* = 56), HeLa cells (*n* = 68), and U2OS cells (*n* = 24) (Fig. 3a). In the DIA (MBR) mode, 2869, 2772, and 1889 protein groups were reproducibly quantified in 80% (i.e. recurrence percentage) of the single A549, HeLa, and U2OS cells, respectively, while only 638, 722, and 1103 protein groups were quantified with less than 20% recurrence percentage (Fig. 3b). These results indicated that the majority of the quantitative identification results of single cells by the PiSPA platform had high levels of reproducibility. In the above single-cell proteomic experiments, a total of 143 and 172 single cells were captured for analysis in the DIA and DDA modes, respectively, and we finally obtained 108 and 148 valid single-cell data, corresponding to average single-cell capture/analysis efficiencies of 76% and 86%.

We implemented a uniform manifold approximation and projection (UMAP) dimensionality reduction on the data of single A549, HeLa and U2OS cells, three types of single-cell samples could be automatically and clearly clustered (Figs. 3d, e), and all of the single cell samples were correctly clustered.

Under the DDA mode, the label-free single-cell protein identification numbers obtained in this work (Fig. 3a) are higher than most of the literature reported results for the same type of cells (e.g. HeLa cells)^12,14,27–29,32^, which shows the advantage of the present workflow and platform in the identification depth of single-cell proteome analysis. Compared with the single-cell proteomic data set obtained under the DIA mode, the single-cell protein identification numbers based on the DDA mode are relatively low. From the unions of the data sets of 37 single A549 cells, 44 single HeLa cells, and 27 single U2OS cells, total numbers of 5093, 5048 and 4286 protein groups could be cumulatively quantified under the DIA mode, respectively (Supplementary Fig. 5). However, only 3286, 3319 and 2357 protein groups were cumulatively quantified from the unions of the data sets of 56 single A549 cells, 68 single HeLa cells and 24 single U2OS cells under the DDA mode, respectively (Supplementary Fig. 5). Under the DIA and DDA modes, the ranges of protein abundance spanned nearly 5 and 4 orders of magnitude, respectively. Some important but low abundant proteins could only be quantified under the DIA mode (Fig. 3c), such as mismatch repair endonuclease PMS2^44^ and E3 ubiquitin-protein ligase NEDD4^45^.

### Evaluation of quantitative accuracy and precision of the LC-MS system

**Fig. 4:**
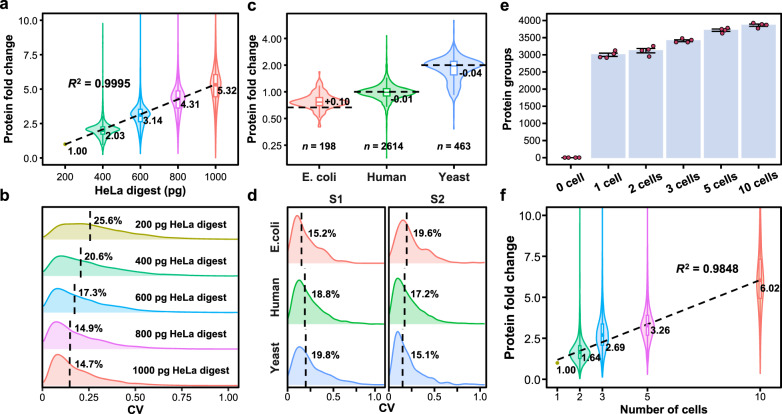
Evaluation of quantitative accuracy and precision of the LC-MS system. **a** Protein abundance fold changes (median = 1.00, 2.03, 3.14, 4.31, and 5.32) for different amounts of HeLa digests (0.2 ng, 0.4 ng, 0.6 ng, 0.8 ng, and 1 ng, *n* = 6) with reference to the average protein abundance in 0.2 ng HeLa digest. **b** Density distribution of coefficient of variation (CV) for proteins in 0.2 ng, 0.4 ng, 0.6 ng, 0.8 ng, and 1.0 ng HeLa digests (median = 25.6%, 20.6%, 17.3%, 14.9%, and 14.7%, *n* = 6). **c** Protein abundance fold changes of *E. coli* (*n* = 198), human (*n* = 2614) and yeast (*n* = 463) measured in the two groups (S2/S1) of the QC samples of digest single HeLa cell spiked with two other species of peptides (S1: single-cell QC added with 50 pg yeast peptides and 150 pg *E. coli* peptides; S2: single-cell QC added with 100 pg yeast peptides and 100 pg *E. coli* peptides; *n* = 4). Dashed black lines indicate the theoretical fold change. **d** CV density distributions of the quantified proteins from the three species in both QC sample groups in (**c**). **e** Quantified protein numbers from 0, 1, 2, 3, 5 and 10 HeLa cells (*n* = 4) under the DIA-MBR mode. The bars indicate the mean values; the error bars indicate the standard deviations; individual data points are presented as red dots. **f** Protein abundance fold changes for 1, 2, 3, 5 and 10 HeLa cells (median = 1.00, 1.64, 2.69, 3.26, and 6.02, *n* = 4) in (**e**), with the average protein abundance of the 1-cell samples as the reference. In (**a**) (**c**) and (**f**), the central lines in the boxes indicate the median values; the boxes indicate the quartiles; the whiskers extend to a maximum of 1.5 times interquartile range beyond the quartiles. Source data are provided as a Source Data file.

To assess the quantitative accuracy and precision of the present LC/MS system, we compared the quantification results of 0.2 ng, 0.4 ng, 0.6 ng, 0.8 ng, and 1 ng HeLa digests (*n* = 6). Using the 0.2 ng HeLa digest as the reference to calculate the protein abundance fold change (FC) values obtained for each quantified protein, a proportionally linear increase in the protein fold change with the increase of the protein amount of the HeLa digests could be observed with *R*^2^ of 0.9995 (Fig. 4a). As the protein amount increased, the quantitative precision improved from a coefficient of variation (CV) of 25.6% with the 200 pg HeLa digest to 14.9% with the1 ng HeLa digest (Fig. 4b). These results demonstrate the favorable quantitative accuracy and precision of the present LC-MS system in the level of single cell and small number of cells.

In addition, we further tested the quantitative accuracy of the system using single cell samples spiked with different ratios of yeast and *Escherichia coli* (*E. coli*) peptides. The QC samples at the single-cell level were prepared by mixing eight digested single HeLa cell samples and then aliquoting it into eight portions. The single-cell QC samples were divided into two groups (S1 and S2) with four samples in each group, and were spiked with yeast peptides and *E. coli* peptides with different ratios to each sample. The spiked QC samples were analyzed with the LC-MS system, and the results showed that the median fold change values of the proteins from the three species in the samples closely matched the theoretical values, with a relative difference, i.e., (median FC - theoretical value)/theoretical value, ranging from –2% to +15% (Fig. 4c). This further demonstrated the high level of quantitative accuracy of the present system. Furthermore, the CV values of the protein quantification results from the three species in both QC sample groups were below 20% (Fig. 4d), also demonstrating the good quantitative precision in the single-cell level.

Benefiting from the ability of the present platform to flexibly and accurately control the cell number in samples, we tested samples containing 0, 1, 2, 3, 5, and 10 HeLa cells with the same procedures and conditions as in single-cell proteomic analysis, to evaluate the background level in the blank control sample as well as examine the effect of cell number on the protein identification number. Under the DIA (MBR) mode, on average, 0, 3000, 3121, 3411, 3718, and 3864 protein groups were quantified in 0, 1, 2, 3, 5 and 10 HeLa cells (*n* = 4), respectively (Fig. 4e). The number of protein identifications did not increase linearly with the cell number (Fig. 4e). However, the protein abundance, as reflected by the median fold changes relative to the single-cell sample, showed a linear relationship with the cell number (Fig. 4f), with FC values of 1.64, 2.69, 3.26 and 6.02 and *R*^2^ of 0.9848 (Fig. 4f). As the “Proteomic Ruler” method^46^, we searched the description of all the quantified proteins in the 1-10 cells HeLa data using the keyword “histone”, and 40 histone-related proteins were obtained. Using the abundances of these proteins in single-cell samples as a reference, the quantitative fold changes (medians) of these proteins in 2, 3, 5, and 10 cells (*n* = 4) was 1.66, 2.30, 3.32, and 6.83, respectively, which was linearly correlated with the number of cells (Supplementary Fig. 6). This further demonstrates the qualified performance of the PiSPA system in terms of cell capture specificity and protein quantification accuracy.

### Single-cell proteomic analysis of migrating cells

Cell migration is a common biological process, which has important significance for the study of tumor migration, wound healing, embryonic development, immune response, etc^47^. At present, the scratch assay is frequently used to test the invasion and metastasis ability of adherent tumor cells^48^, while so far there is no report on the study of individual tumor cells with different migration behaviors at the deep-coverage proteome level. The PiSPA platform has the ability to observe the behavior of individual migrated cells, reliably pick the target cells up, and achieve deep-coverage proteomic analysis to these cells at the single-cell level to highlight the individual protein differences between cells with different apparent migratory properties.

**Fig. 5:**
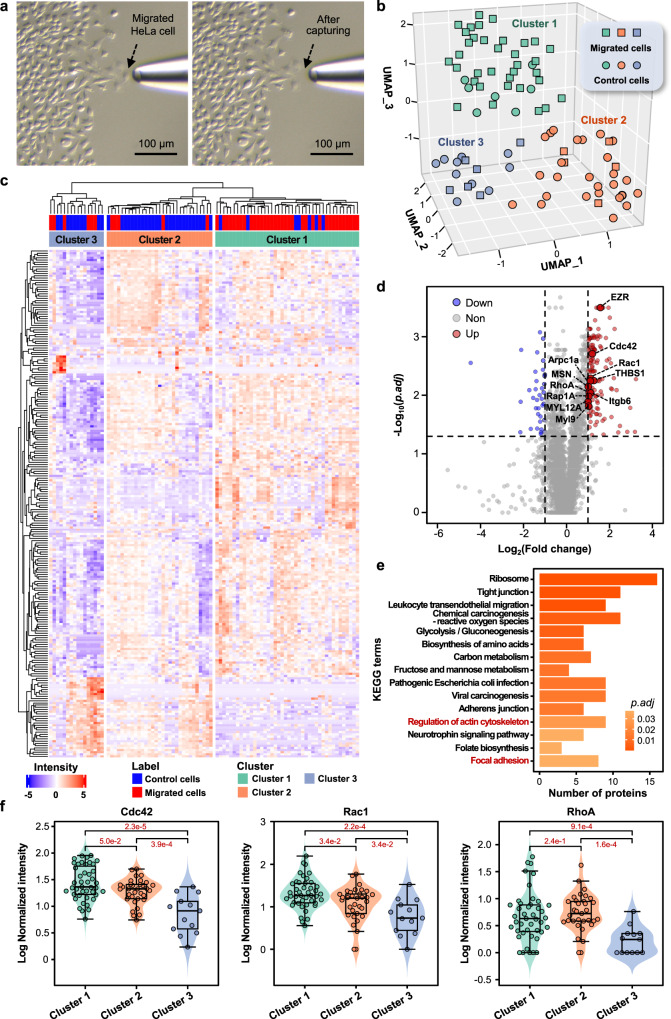
Application of the PiSPA platform for proteomic analysis of single migrated cells in scratch assay. In the scratch assay, 46 single HeLa cells with significant migration behaviors and 43 single HeLa cells without obvious migration behaviors as control cells were captured and analyzed in the experiment of scratch assay. Conditions: enzyme/protein ratio, 50:1; LC gradient time, 21 min. **a** Micrographs showing the target-cell picking-up operation in the scratch assay before and after the capturing of the migrated HeLa cell (*n* = 46) by the PiSPA platform. **b** 3D UMAP clustering analysis of the tested migrated (square) and control (circle) HeLa cells, being clustered into in cluster 1 (cyan), cluster 2 (orange) and cluster 3 (blue). **c** Hierarchical clustering heatmap of 226 differential proteins in the three clusters in (**b**). **d** Volcano plot showing 135 up-regulated proteins and 36 down-regulated proteins screened out in cells of cluster 1 compared to cluster 3, including 11 proteins involved in signal pathways of focal adhesion and regulation of actin cytoskeleton. **e** KEGG enrichment analysis of the differential proteins between the cells of cluster 1 and cluster 3. Pathways of “focal adhesion” and “regulation of actin cytoskeleton” are marked in red. The enrichment test *p* values were adjusted using Benjamini-Hochberg correction. **f** Comparisons of the quantitative expression levels of Cdc42, Rac1 and RhoA proteins in cells of cluster 1 (*n* = 42), cluster 2 (*n* = 31) and cluster 3 (*n* = 16). The central lines in the boxes indicate the median values of the corresponding data; the boxes indicate the quartiles; the whiskers extend to a maximum of 1.5 times interquartile range beyond the quartiles; individual data points are overlaid. The adjusted *p* values are indicated in the figure. In (**c**, **d**, **f**), the differential proteins were determined by fold change >2 and adjusted *p* value < 0.05 (two-side Wilcoxon test with Benjamini-Hochberg correction). Source data are provided as a Source Data file.

We used HeLa cells to perform the scratch assay, and 46 single cells with significant migratory behaviors (Fig. 5a, Supplementary Data 1) and 43 single (control) cells without obvious migratory behaviors were captured and analyzed with the PiSPA platform. Under the DIA (MBR) mode, an average of 2544 (2058–3308) and 2893 (1896–3710) protein groups were quantified from single migrated cells (*n* = 46) and control cells (*n* = 43), respectively (Supplementary Data 2, Supplementary Data 3). Among them, over 78% of the proteins were quantified by two or more feature peptides (Supplementary Fig. 7).

After batch correction and normalization, the data were clustered into three distinct clusters by UMAP (Fig. 5b). Cluster 1 (*n* = 42) predominantly consisted of migrated cells (*n* = 35, 83%), while cluster 2 (*n* = 31) and cluster 3 (*n* = 16) consisted mainly of control cells (*n* = 26 and *n* = 10, 83% and 63%, respectively). By individually comparing the three clusters, 226 significantly different proteins were screened out (Wilcoxon test, *p.adj* < 0.05, fold change >2). Hierarchical cluster analysis (HCA) was utilized to quantitatively assess the differences in protein compositions among the three clusters (Fig. 5c) to feature the heterogeneity among the different clusters.

Compared to cluster 2, there were 33 proteins significantly upregulated and 13 proteins significantly downregulated in cluster 1 (Supplementary Fig. 8, Wilcoxon test, *p.adj* < 0.05, fold change > 2). Among them, the upregulated proteins such as Orc1^49^, TGFBI^50^, MSLN^51^, HtrA1^52,53^ were reported to be associated with the migration and invasion of tumor cells. The expression levels of these proteins in the different clusters were quantified (Supplementary Fig. 9), and it was observed that these proteins exhibited significantly downregulated in both cluster 2 and cluster 3, which corresponded to the relatively weak apparent migration behavior of most of the cells in the two clusters.

We further compared the protein expression differences between cluster 1 and cluster 3. In cluster 1, there were 135 proteins exhibiting upregulation and 36 proteins showing downregulation (Fig. 5d, Wilcoxon test, *p.adj* < 0.05, fold change >2). Among them, 11 upregulated proteins, including Cdc42, Rac1, RhoA, EZR, THBS1, Itgb6, Rap1A, MYL12A, Myl9, MSN, and Arpc1a, were involved in signal pathways of focal adhesion and regulation of actin cytoskeleton (Fig. 5e, Kyoto Encyclopedia of Genes and Genomes (KEGG)^54^ enrichment analysis, *p.adj* < 0.05), which were reported to be closely related to cell migration^55,56^. In the previously-reported cell migration models^57–60^, the function of Cdc42 is usually recognized as inducing filopodia formation and regulating the direction of cell migration, the function of Rac1 is to induce lamellipodia formation and establish new adhesion sites at the leading edge to pull the cell forward, and the function of RhoA is to promote the contraction of the posterior actin to detach from adhesions, eventually leading to the migration of cells. We clearly observed a significant upregulation of these above-mentioned proteins in cluster 1 compared to cluster 3, implying the activation of the relevant migration pathways, which was in agreement with the fact that the cluster 1 cells exhibited significant apparent migration property in the migration assay. Interestingly, we compared the expression levels of the three proteins at the single-cell level in the three clusters (Fig. 5f) and the results showed that the expressions of the three proteins in cluster 2 cells were also higher than those in cluster 3, showing obvious heterogeneity among the control cells. This result implied that although most cells in both cluster 2 and cluster 3 did not show significant migration behaviors, probably the reasons for their lack of significant migration behavior were different. The upregulations of the migration-associated proteins in cluster 2 cells implied the potential strong migration activity of these cells, while they did not show actual migration behaviors presumably due to the lack of space around these cells for migration. Such a cell protein heterogeneity that exists within a cell group with similar phenotypic behaviors is difficult to observe and explore with the conventional bulk cell experiments except using the single-cell proteomic analysis technique.

The use of the “pick-up” approach enabled the observation of evident cellular heterogeneity in cell migration, such as the presence of a small number of mixed cells within the same clusters. However, limited by the relatively rough operation mode of the scratch experiment in the condition control and monitoring of cell migration, the current experimental data could not provide a definitive explanation for these types of cellular heterogeneity, implying the possible existence of more complex relationships between cell migration behaviors and protein expression. For further validation and in-depth study on these relationships in the future, microfluidic chips could be used to precisely control the position and state of the initial cells and a live cell workstation could be used to monitor the whole migration process for tracking the migration paths of each tested cells.

The present application of single-cell proteomics in cell migration experiment is just a preliminary proof-of-principle attempt, while it could demonstrate that the PiSPA platform has the potential to reveal the intrinsic control factors behind the apparent behaviors that were previously difficult to detect using the conventional omics approaches for large amounts of cell samples, and could provide an effective tool for the cell migration studies as well as the development of anticancer approaches and drugs.

## Discussion

In the present work, we adopted the single-cell customization strategy throughout the whole process of the platform building, methodology development and performance improvement for single-cell proteomic analysis, covering workflows of single-cell sorting, sample pretreatment, chromatographic injection and separation, mass spectrometry detection and data processing. A total solution was developed to achieve a leap improvement in protein identification depth and reliable and convenient operation of single-cell analysis. The PiSPA platform achieved single-cell sorting using the pick-up mode, which is different from the flow cytometry^3^, CellenONE^61^, or limiting dilution based^13,62^ single-cell sorting approaches. Under the pick-up mode, arbitrary cells of interest can be selected as the target cells in the microscopic field of view based on the bright-field or fluorescence information or other properties of the cells, and the target cells are picked individually by the capillary probe under microscopic monitoring. Therefore, this approach has strong controllability, definiteness and reliability in the selection and picking of the target cells, while retaining the phenotypic and spatial information of these target cells. This feature has been demonstrated in the picking of migrated cells in the cell migration experiment, which is difficult to achieve using the flow cytometry-based single-cell sorting techniques. Although the throughput (~20 s per cell) of the PiSPA platform in single-cell sorting is far lower than those of the flow cytometry-based systems, it can still match the current throughput of tens of samples per day in most of single-cell proteomic analysis systems. If needed, the cell sorting throughput could be further increased multiplicatively by using an array of capillary probes.

The PiSPA platform integrated the microfluidic liquid handling robot with the insert tube array to achieve the loading of single-cell droplets and subsequent multi-step nanoliter-scale sample pretreatment, using the robot’s pick-up operation and the conical bottom tips of the insert tubes as the nanoliter reaction vessels. Compared with the microchip-based reactors fabricated with special equipment and complex procedures^13,16–18,25,30,31,35^, the insert tubes have the merits of low-cost, easily available, and convenient to use. In addition, such an arrangement also enabled the seamless integration of single-cell sorting, nanoliter-scale sample pretreatment and subsequent automated liquid chromatography injection of the samples, which significantly improved the operability, reliability and success rate of the whole workflow of single cell proteomic analysis. This is of significance for promoting the practical popularization of single-cell proteomic analysis.

Considering that the conventional proteomic analysis of samples containing thousands of cells can typically identify about 5000–7000 protein groups^8,18^, the PiSPA platform has the ability to quantify nearly half this level for a single mammalian cell with a significant improvement over many current single-cell proteomic analysis systems. This may imply that the single-cell proteomics research has entered a stage of practical application in a wide range of biomedical research fields.

Looking back at the breakthrough in identification depth of single-cell transcriptome sequencing technology ten years ago^63–66^, the Smart-seq-based single-cell RNA-seq technique could identify around 30,000 transcripts from one human mammalian cell^63^, accounting for 13% of the total human transcripts (ca. 230,000^67^). In this study, up to 3,000 proteins quantified in one human mammalian cell account for 15% of the total human proteins (ca. 20,000^67^), reaching the similar level of single-cell RNA-seq technique at that time. Considering the explosive development of the single-cell transcriptome sequencing technology over the past decade^68–70^, we may now be on the eve of or even in the outbreak of the single-cell protein sequencing technology based on the shotgun proteomics strategy.

In the near future, the identification depth and throughput of single-cell proteomic analysis will be further improved, reaching the level of practical and popular application. In addition, by adopting the microfluidic sample pretreatment technique, it will be possible to combine it with single-cell genome, transcriptome, and metabolome analysis technologies to form a “true” single-cell multi-omics analysis technology for single-cell individuals. These will undoubtedly bring unprecedented powerful tools for people to understand the variations of cellular heterogeneity in life activities.

## Methods

### Cell culture

HeLa (SCSP-504), A549 (SCSP-503) and U2OS (SCSP-5030) cells were purchased from the Cell Bank of the Chinese Academy of Sciences. The HeLa and U2OS cells were maintained in Dulbecco’s modified eagle medium (DMEM) with high glucose/pyruvate (Invitrogen) supplemented with 10% fetal bovine serum (FBS) (Gibco). The A549 cells were maintained in F-12K supplemented with 10% FBS. All cell lines were maintained in a 5% CO_2_ incubator at 37 °C. For single-cell analysis, cells in a 6 cm dish were collected at 60–80% confluency using 0.25% trypsin with 0.02% EDTA, and washed five times with phosphate buffer solution (PBS). All cell lines were authenticated by STR profiling (BIOWING, Shanghai). No positive sign of mycoplasma contamination for all cell lines.

### Single cell capture

The microfluidic liquid handling robot integrated in the PiSPA platform was developed based on a SODA system for single-cell soring^37^. The SODA technique was first developed by the authors’ group in 2013 for achieving automated picoliter to nanoliter liquid manipulation^38,39^ and has been applied in high-throughput screening, single cell analysis^13,37,39^, microscale cell assays, and micro-sample analysis. The liquid handling robot consisted of a microscopic imaging module for cell observation and identification, a capillary probe (10 cm length, 250 μm o.d., 150 μm i.d., tip size, 35 μm i.d.) connected with a high-precision syringe pump (1701N, Hamilton) for nanoliter-scale liquid metering/handling, an array of insert tubes (4 mm inner diameter, 31 mm height, 300 μL volume, ALWSCI Co.) for loading nanoliter-scale single-cell droplets and reaction solutions, an automated x-y-z translation stage for controlling the movement of other modules, and a system control module for the whole robot under the control of a computer program. In addition to cell sorting, the function of the robot was extended to complete nanoliter-scale 5-step single-cell sample pretreatment reactions. Instead of droplet array chips^13^ frequently used in the previous SODA systems, we used the array of insert tubes with their conical bottom tips as nanoliter reactors to improve the operability and reliability of the system as well as to facilitate the subsequent automated liquid chromatography injection. Based on this improved robotic system, we proposed the pick-up mode-based automated cell sorting and sample pretreatment approach, whereby the whole workflow of deep single-cell proteomic analysis was established.

For picking up target cells, 2 mL of cell PBS suspension was added in a petri dish (35 mm diameter) fixed on the translational stage of the robot. Usually, the cell dispersion density at the bottom of the culture dish was controlled to be less than 10,000 cells/cm^2^ (corresponding to a cell suspension density of <100,000 cells/mL), for ensuring the sufficient distances between the target cells and the surrounding adjacent cells (usually >30 μm) to avoid the adjacent non-target cells to be sucked into the capillary probe. The microscopic imaging module first took bright-field or fluorescence image of the cells in the cell suspension settled on the bottom of the petri dish. The target cells in the image were selected based on their bright-field apparent property or labeled fluorescence signals, and their location coordinates were automatically calculated by the control module. Then the tapered tip of the capillary probe was controlled to automatically align the target cell by moving the translational stage and suck it into the probe by aspirating 15 nL of cell suspension into the probe by the syringe pump. Before the cell picking-up operation, the capillary probe was prefilled with 50 mM NH_4_HCO_3_ solution. After the target cell was picked up by the capillary probe, it was then deposited to the bottom of an insert tube by dispensing 400 nL of the solution in the probe. Since the present pick-up mode employed the procedure of picking one target cell at one time and immediately dispensing it into the insert tube, and the capillary tip had an inner diameter of 35 µm which was larger than the cell size, capillary blockage was rarely encountered during the cell picking and dispensing process.

### Single-cell sample pretreatment

After picking up the single target cell into the insert tube, we used the robot to perform the subsequent sample pretreatment operations, including cell lysis, protein reduction, alkylation, enzymatic digestion and termination of the digestion reaction. First, 100 nL of 0.3% (w/v) RapiGest SF (Waters) solution was added to the insert tube, and the insert tube was inserted into a sample vial with a sealing cap and 100 μL of water prefilled in the vial. Multiple sample vials were formed an array of vials, which was heated in an oven for 20 min at 60 °C to lyse the cells. After the sample vials were cooled to room temperature, 100 nL of 20 mM tris (2-carboxyethyl) phosphine (TCEP) was added to each insert tube, for performing the protein reduction reaction for 20 min at room temperature, and 100 nL of 125 mM iodoacetamide (IAA) was added to conduct the alkylation reaction to the protein cysteine residues at room temperature for 15 min in dark. Then, 100 nL of a mixed enzyme (0.05 μg/μL Lys-C and 0.05 μg/μL trypsin) solution was added to the insert tube to digest the proteins for 2 h at 37 °C. Finally, 100 nL of 40% formic acid (FA) solution was added to terminate the enzymatic reaction with an incubation time of 30 min at room temperature. To avoid cross-contamination between different reagents and samples, the capillary probe was washed with water three times before aspirating new reagents or samples. The sample vials with the insert tubes were placed in the sample tray of the LC autosampler for LC-MS/MS analysis.

During the single-cell dispensing and reagent addition processes, the caps of the sample vials were lifted and the sample vials were in open state to facilitate the capillary probe inserting into the insert tube for performing the relevant operations. Since these single-cell dispensing and reagent addition operations took only 5-10 s per tube and the sample solutions in the insert tubes were protected by the large volume of water added to the sample vials, the evaporation of hundreds of nanoliters of sample solutions during these operations was at a negligible level. In the reaction incubation and chromatographic injection processes, the sample vial caps were in a sealed closed position to avoid the significant evaporation losses of the sample solutions during prolonged processes of heating incubation or waiting in line for LC injection. In addition, the sample vial caps were installed with a seal membrane in each cap that could be penetrated by the sampling probe in the autosampler to allow it to insert into the single-cell sample solutions for sampling and LC injection.

For the condition optimizing experiments (Fig. 2), 24 HeLa cells were analyzed for the optimization of the enzyme/protein ratio (3 cells for each of the 8 conditions), and 18 HeLa cells for the optimization of the LC gradient time (3 cells for each of the 6 conditions). For the single-cell analysis (Fig. 3), 256 cells were analyzed (37 A549, 44 HeLa and 27 U2OS cells in DIA mode; 56 A549, 68 HeLa and 24 U2OS cells in DDA mode).

### Preparation of quality control samples

Commercial HeLa protein digest powders (Thermo Scientific) were used to prepare standard HeLa digests at concentrations of 0.2 ng/μL, 0.4 ng/μL, 0.6 ng/μL, 0.8 ng/μL, and 1.0 ng/μL, which served as quality control (QC) samples for evaluating the performance of the LC-MS system. For the condition optimizing experiments (Fig. 2), 20 QC samples (0.2 ng/μL) were analyzed for LC-MS repeatability evaluation (10 samples for DDA and 10 samples for DIA). For the quantitative accuracy and precision evaluation (Fig. 4), 30 QC samples were analyzed (6 samples for each of the 5 concentrations). Two QC samples were used to evaluate the effect of RapiGest SF on single cell proteomic analysis (Supplementary Fig. 10).

The QC samples at the single-cell level were prepared by mixing eight digested single HeLa cell samples and then aliquoting it into eight portions. The single-cell QC samples for testing the quantitative accuracy of the system were divided into two groups (S1 and S2) with four samples in each group. For the S1 group, 50 pg of yeast peptides and 150 pg of *E. coli* peptides were added to each sample; for the S2 group, 100 pg of yeast peptide and 100 pg of *E. coli* peptide were added to each sample. For the quantitative accuracy and precision evaluation (Fig. 4), 8 mixed-species samples (4 samples for each of the 2 conditions) and 24 single/multi-cell samples (4 samples for each of the 6 conditions) were analyzed (Supplementary Fig. 11).

### Cell migration experiment

The cell migration experiment was performed using the scratch assay mode. A scratch (blank area) was first drawn by a 1-mL pipette tip on a densely growing monolayer of HeLa cells in a petri dish. After washing with PBS, a new culture medium was added to the petri dish and no cells could be observed in the blank area. After 24-h culture at 37 °C, a small number of cells migrated into the blank area. Utilizing the ability of the liquid handling robot in picking up each target cell accurately and reliably, we employed it to pick up 46 cells in the blank area and 43 cells (control cells) outside the blank area for single-cell proteomics analysis.

### LC-MS/MS analysis

A capillary LC column (10 cm length, 360 o.d., 50 μm i.d.) packed with 1.7 μm C_18_ particles (120 Å pore size, Nanomicro Co.) was used in the LC separation of the digested peptide samples. The single-cell samples in the insert tubes were injected by an autosampler coupled to an EASY-nLC 1200 LC (ThermoFisher Scientific). Mobile phases (A, 0.1% formic acid in water; B, 0.1% formic acid, 80% ACN in water) with a 21 min gradient (0–13 min, 3–40% B; 13–14 min, 40–100% B; 14–21 min, 100% B) at a flow rate of 150 nL/min was used for the separation of single-cell samples.

The separated peptide components of single cell samples were detected by a trapped ion mobility-time of flight mass spectrometer (timsTOF Pro, Bruker). Data dependent acquisition (DDA) mode and data independent acquisition (DIA) mode were used in the MS data acquisition of both the QC sample and single-cell samples to compare their performance in single cell analysis, since it was reported previously that the DIA mode generally has an advantage in protein identification depth over the DDA mode under the same LC conditions^19^. For DDA mode, the range of the ion mobility 1/k0 was 0.75–1.3, and the parallel accumulation serial fragmentation (PASEF) acquisition mode was used. The *m/z* acquisition range was 300–1500, the spray voltage was 1750 V, and the ion accumulation time was 166 ms. The precursor ions obtained by the primary mass spectrum underwent secondary fragmentation through collision induced dissociation (CID). The size of the precursor ion isolation window was related to the ion *m/z*. For ions with *m/z* below 700, the separation window was 2, for ions with *m/z* above 800, the separation window was 3. The dynamic rejection time was 0.4 min, and the CID collision energy was in the range of 20–59 eV, which varied with ion mobility. To obtain the optimal MS detection under the DIA mode, we tested the acquisition range of *m/z* = 384-1171 and the isolation window of *m/z* = 20-40 with both samples of 200 pg HeLa digest and single HeLa cells, and finally selected the optimized *m/z* acquisition range of 399-1124 and isolation window of *m/z* = 25. In addition, we optimized the ramp time to be 166 ms, to accommodate the low protein input at the single cell level.

### MS data analysis

Mass spectrometry data was collected using Compass Hystar software (version 5.1). The DDA raw files were analyzed with SpectroMine software (version3.2, Biognosys AG, Schlieren, Switzerland) using default settings. Protein label-free quantitative identification from the DDA dataset was searched against the UniProt Proteomes *Homo sapiens* database (accession: UP000005640, taxon ID: 9606, 20,626 entries, access date 2021-01). Peptides with a length of 7 to 52 amino acids were considered for the search. Enzyme specificity was set to trypsin cleaving C-terminal to arginine and lysine. A maximum of one missed cleavage were allowed. Cysteine carbamidomethylation was set as a static modification, acetylation on protein N-terminus and oxidation on methionine set as variable modifications. The peptide-spectrum matches (PSM), peptides and protein groups were filtered at 1% false discovery rates (FDR). The DIA raw files were analyzed with DIA-NN^71^ software (version 1.8) in library-free search mode using default settings. The DIA raw data files for single cells were searched against UniProt Proteomes *Homo sapiens* database. The DIA raw data files of single-cell QC spiked with two species were searched against UniProt Proteomes *Homo sapiens*, Swiss-Prot *Escherichia coli* (strain K12) (taxon ID: 83333, 4530 entries, access date 2023-07) and *Saccharomyces cerevisiae* (strain ATCC 204508 / S288c) (taxon ID: 559292, 6727 entries, access date 2023-07) database. Peptides with lengths of 7 to 52 amino acids were considered for the search, and the Q-values were controlled at 1% on precursor and protein group level.

The pairwise correlation analysis between the protein quantification data of the QC samples were performed by Python (version 3.9.15) and the package Pandas (version 1.4.4). The fold change in protein abundance and density distribution of coefficient of variation were analyzed by R (version 4.1.3). Proteins with missing values ≥ 50% were excluded from the CV calculation. Forty histone-related proteins were searched out among the quantified proteins from multiple-cell HeLa data with the keyword “histone”.

### Bioinformatics analysis

Single-cell proteomics data were analyzed by R (version 4.1.3) and the R package Seurat^72^ (version 4.3.0). Proteins quantified in <3 cells were excluded and missing values were imputed with zeros. The protein quantities were normalized to sum to 10,000 for each cell, and then transformed to natural logarithm. Batch effects of samples were corrected by the R package harmony^73^ (version 0.1.1). Dimensional reduction was performed by principal components analysis (PCA) and the top 25 principal components were used for the uniform manifold approximation and projection (UMAP) analysis. The cells were divided into 3 clusters using the Louvain binning algorithm (Supplementary Fig. 12, Supplementary Note 1). The protein quantities were compared between clusters pairwisely, where only proteins with no more than 75% missing values across all the compared cells were taken into consideration. Differential proteins were determined by the Wilcoxon test (p.adj <0.05 by the Benjamini-Hochberg method, fold change > 2). Kyoto Encyclopedia of Genes and Genomes (KEGG)^45^ enrichment was carried out by the R package clusterProfiler^74^ (version 4.8.1).

The 2D-UMAP of the tumor cells was visualized by Python and the package umap-learn (version 0.5.3). The 3D-UMAP in the cell migration study was visualized by the R package plot3D (version 1.4), and the heatmap was visualized by the R package ComplexHeatmap^75^ (version 2.16.0). Other data were visualized by the R package ggplot2 (version 3.3.5).

### Reporting summary

Further information on research design is available in the Nature Portfolio Reporting Summary linked to this article.

### Supplementary information

Supplementary information

Description of Additional Supplementary Files

Supplementary Data 1

Supplementary Data 2

Supplementary Data 3

Reporting Summary

### Source data

Source data

## Data Availability

The mass spectrometry proteomics data generated in this study have been deposited to the ProteomeXchange Consortium via the iProX partner repository^76,77^ with the dataset identifier PXD041966 or IPX0006351000. *Homo sapiens* (accession: UP000005640, taxon ID: 9606, 20,626 entries, access date 2021-01), *Escherichia coli* (strain K12) (taxon ID: 83333, 4530 entries, access date 2023-07) and *Saccharomyces cerevisiae* (strain ATCC 204508 / S288c) (taxon ID: 559292, 6727 entries, access date 2023-07) protein databases were downloaded from UniProt [https://www.uniprot.org], and have also been deposited to the ProteomeXchange/iProX repository. Source data are provided with this paper.
