# Supplementary material for: Pick-up single-cell proteomic analysis for quantifying up to 3000 proteins in a Mammalian cell
Source: Nat Commun. 2024 Feb 10;15:1279. doi: 10.1038/s41467-024-45659-4 (PMC10858870; doi:10.1038/s41467-024-45659-4)
Supplement: Supplementary file 1 — Supplementary information [file 41467_2024_45659_MOESM1_ESM.pdf]

Supplementary Information

Pick-up single-cell proteomic analysis for quantifying up to 3000 proteins in a tumor cell

Yu Wang[1,2,3,#], Zhi-Ying Guan[1,#], Shao-Wen Shi[2], Yi-Rong Jiang[1], Jie Zhang[4], Yi Yang[1,2], Qiong Wu[1], Jie Wu[1], Jian-Bo Chen[1], Wei-Xin Ying[1], Qin-Qin Xu[1], Qian-Xi Fan[1], Hui-Feng Wang[2], Li Zhou[5], Ling Wang[5], Jin Fang[4], Jian-Zhang Pan[1,2] and Qun Fang[1,2,6] ✉

[1] Institute of Microanalytical Systems, Department of Chemistry, Zhejiang University, Hangzhou 310058, China.

[2] Single-cell Proteomics Research Center, ZJU-Hangzhou Global Scientific and Technological Innovation Center, Hangzhou 311200, China.

[3] College of Computer Science and Technology, Zhejiang University, Hangzhou 310027, PR China.

[4] Department of Cell Biology, China Medical University, Shenyang 110122, China.

[5] Shanghai Omicsolution Co., Shanghai 201100, China.

[6] Key Laboratory of Excited-State Materials of Zhejiang Province, Zhejiang University, Hangzhou 310007, China.

[#] These authors contributed equally: Yu Wang, Zhi-Ying Guan.

✉e-mail: fangqun@zju.edu.cn

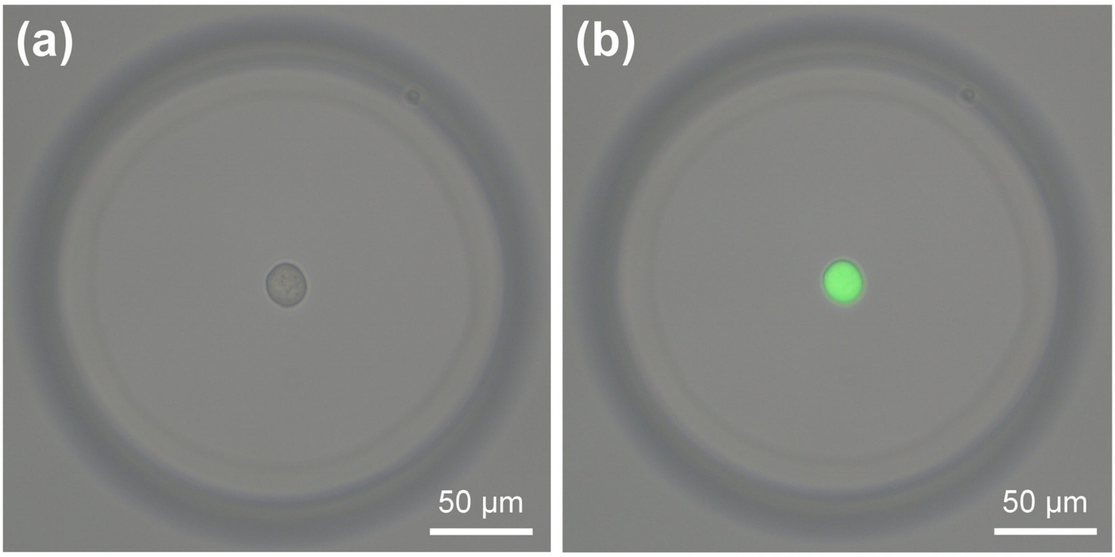

Supplementary Figure 1. Typical microscopic images of a picked HeLa cell in a droplet after staining by the fluorescent dye for cell viability assay. **(a)** bright field image; **(b)** fluorescence image. The HeLa cell was picked and dispensed into a 20 nL PBS droplet by the PiSPA platform. The image in **(a)** shows the cell had an intact morphology and could be attached to the bottom surface of the vessel. The fluorescence image in **(b)** shows the cell could retain its viability after staining by the fluorescent dye.

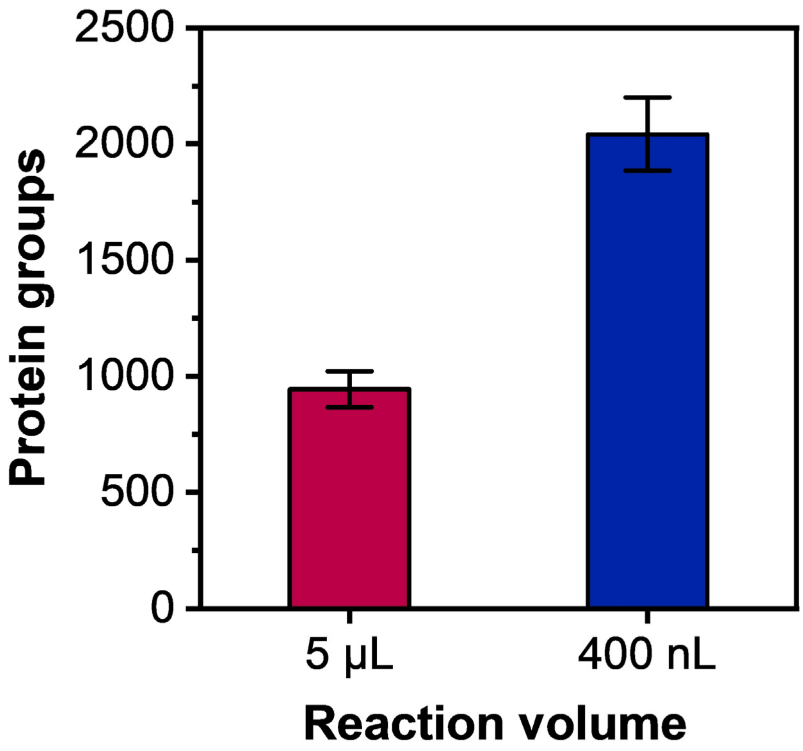

Supplementary Figure 2. Comparison of the protein group number quantified in single HeLa cells with microliter-scale and nanoliter-scale reaction volumes for sample pretreatment. In average, 944 ± 77 (n = 4) and 2042 ± 158 (n = 4) protein groups were quantified under the DDA mode with the reaction volumes of 5 μL and 400 nL, respectively. The bars indicate the mean values of the corresponding data, and the error bars indicate the standard deviations. Source data are provided as a Source Data file.

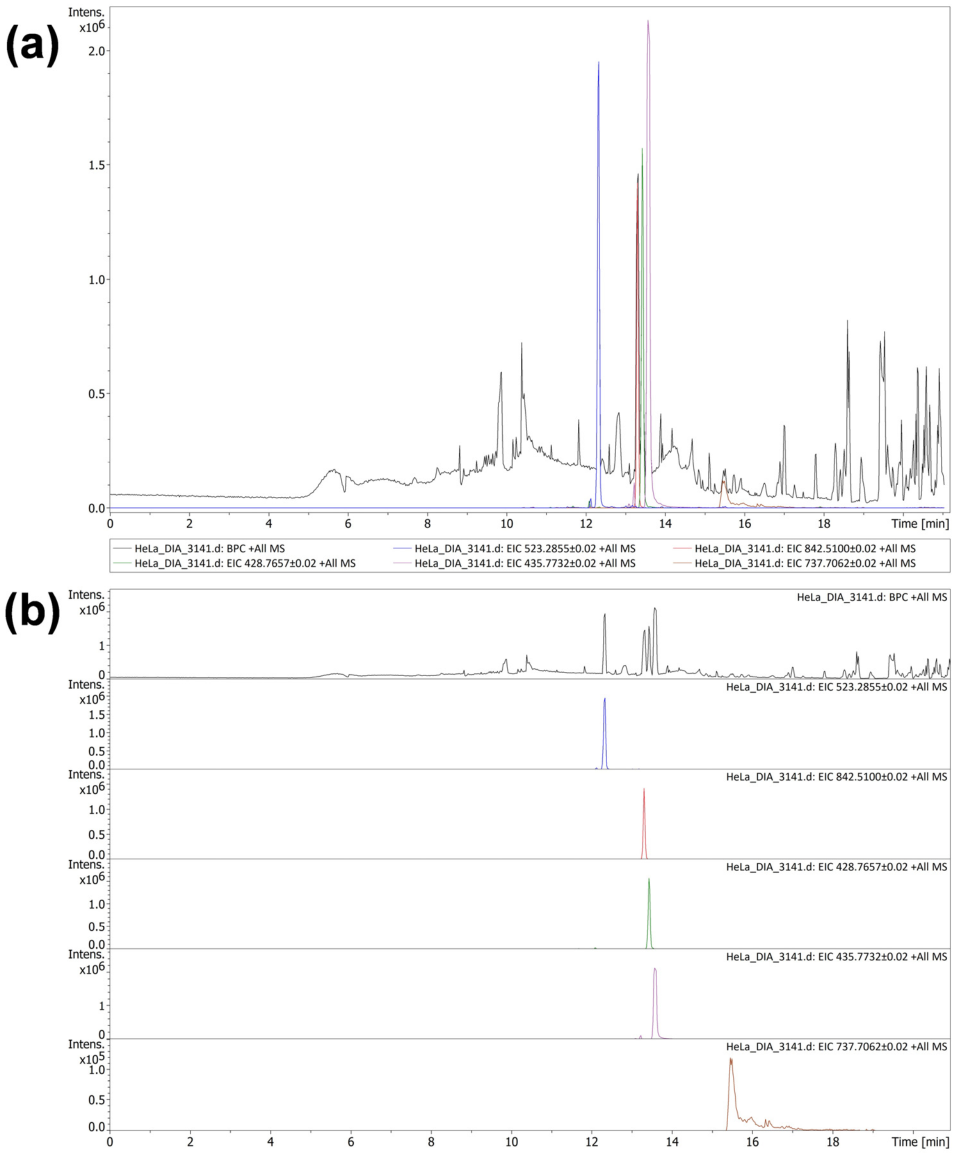

Supplementary Figure 3. Evaluation of the effect of self-cleavage of trypsin. The typical base peak chromatogram (BPC, black line) of a single HeLa cell sample in proteomic analysis along with the extracted ion chromatograms (EIC, colored lines) of the identified self-cleaved peptides of trypsin are shown in an overlapping format **(a)** and in a list format **(b)**. The extracted self-cleaved peptides include LSSPATLNSR 2+ (*m/z* = 523.2855), VATVSLPR 1+ (*m/z* = 842.5100), VATVSLPR methylated 2+ (*m/z* = 428.7657), VATVSLPR dimethylated 2+ (*m/z* = 435.7732), and LGEHNIDVLEGNEQFINAAK 3+ (*m/z* = 737.7062). It was observed that the signals of the self-cleaved peptides of trypsin appeared at only a few specific retention time and *m/z* positions and had no evident effect on the identification of the sample peptides.

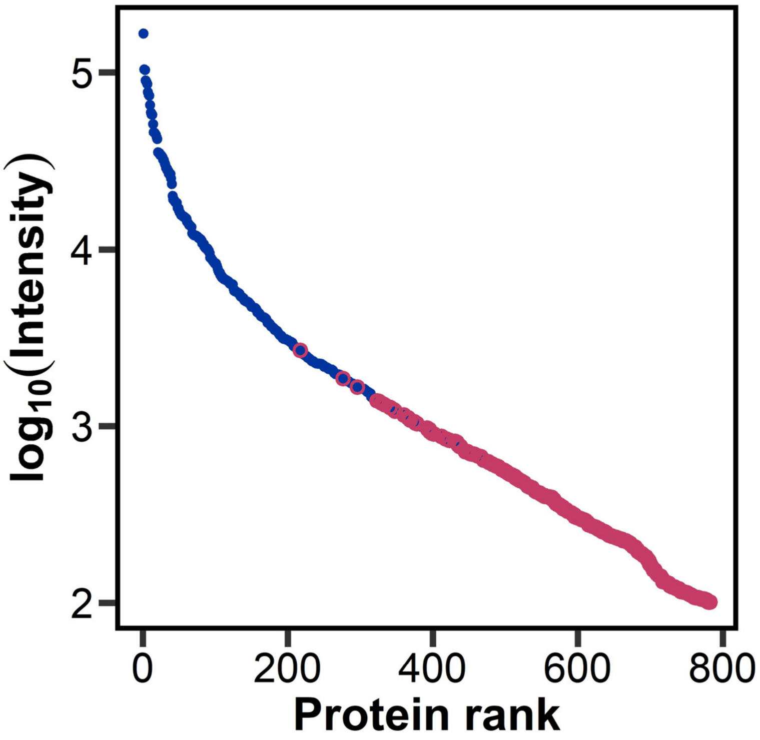

Supplementary Figure 4. Comparison of the protein group number quantified in single HeLa cells with chromatographic gradients of 21-min and 68-min. The standard sample containing 200 pg of tryptic peptides from HeLa cell digestion was used. A total of 782 and 549 protein groups (n = 3) were quantified under the DDA mode with chromatographic gradients of 21 and 68 min, respectively. The proteome quantified with the 21-min and 68-min gradient were ranked according to the average identification intensity. The blue dots were used to indicate the proteins that could be quantified under both the 21-min and 68-min gradient, while the red dots were used to indicate the proteins that only be quantified under the 21-min gradient. The proteins quantified specifically under the 21-min gradient are mainly low-abundance proteins. Source data are provided as a Source Data file.

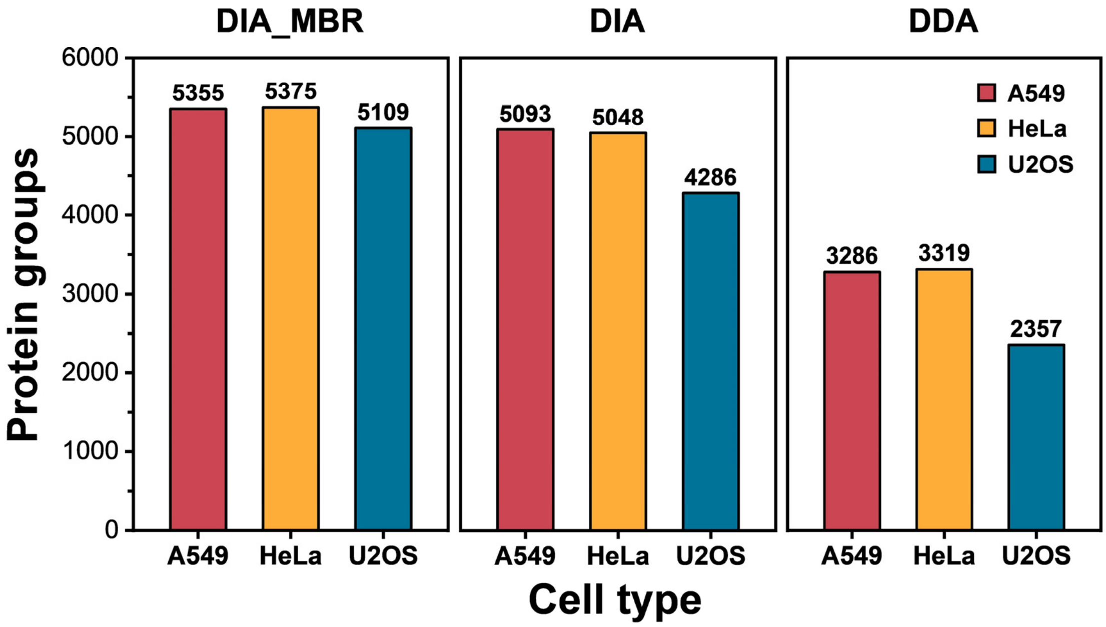

Supplementary Figure 5. Cumulative total numbers of protein groups quantified in the samples of single A549, HeLa and U2OS cells. From the unions of the data sets of 37 single A549 cells, 44 single HeLa cells, and 27 single U2OS cells, and total numbers of 5355, 5375 and 5109 protein groups were quantified cumulatively under the DIA mode with MBR, respectively, and total numbers of 5093, 5048 and 4286 protein groups were cumulatively quantified under the DIA mode, respectively. From the unions of the data sets of 56 single A549 cells, 68 single HeLa cells, and 24 single U2OS cells, total numbers of 3286, 3319 and 2357 protein groups were quantified cumulatively under the DDA mode, respectively. Source data are provided as a Source Data file.

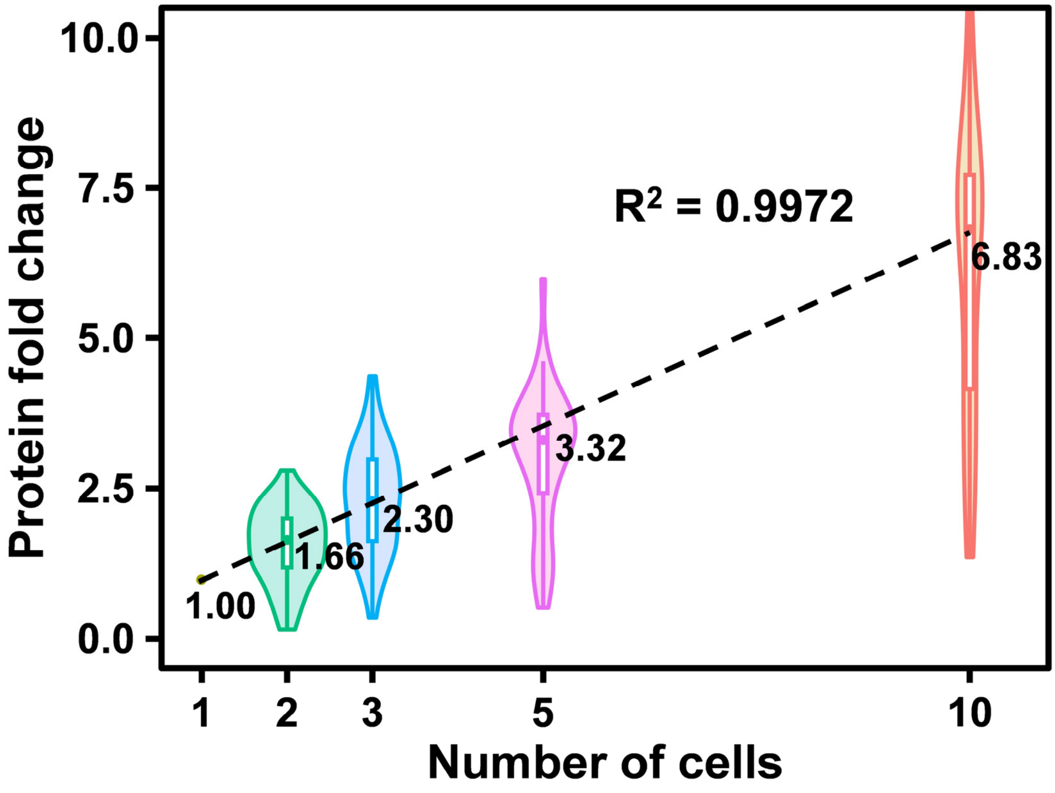

Supplementary Figure 6. Fold changes of histone-related protein abundance for 1, 2, 3, 5 and 10 HeLa cells (median = 1.00, 1.66, 2.30, 3.32, 6.83, $n = 4$), with the average abundance of histone-related proteins ($n = 40$) in the single-cell sample as the reference. The central lines in the boxes indicate the median values; the boxes indicate the quartiles; the whiskers extend to a maximum of 1.5 times interquartile range beyond the quartiles. Source data are provided as a Source Data file.

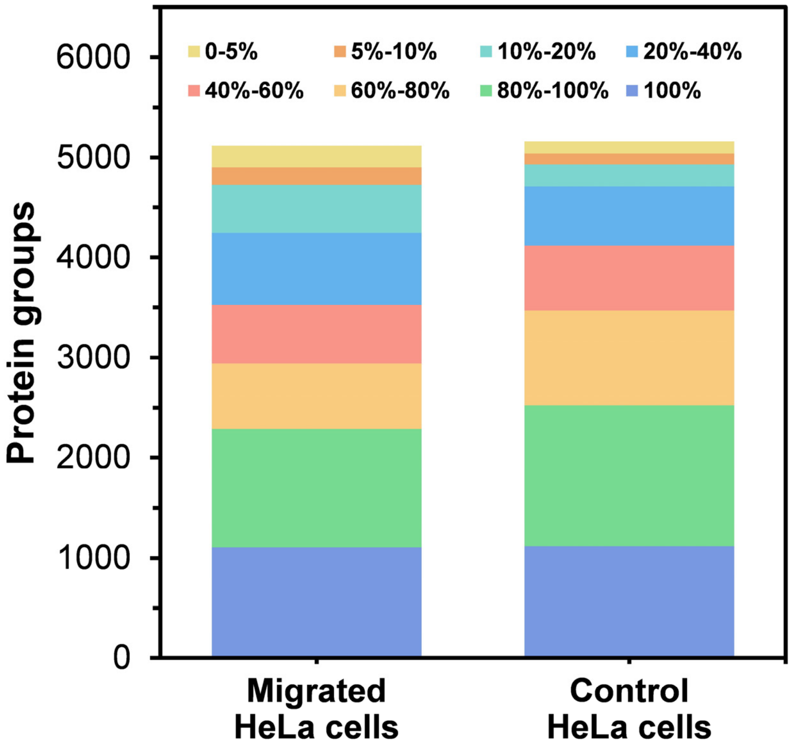

Supplementary Figure 7. Recurrence percentage distributions of the protein groups quantified in the migrated and control HeLa cells in the scratch assay under the DIA-MBR mode. Source data are provided as a Source Data file.

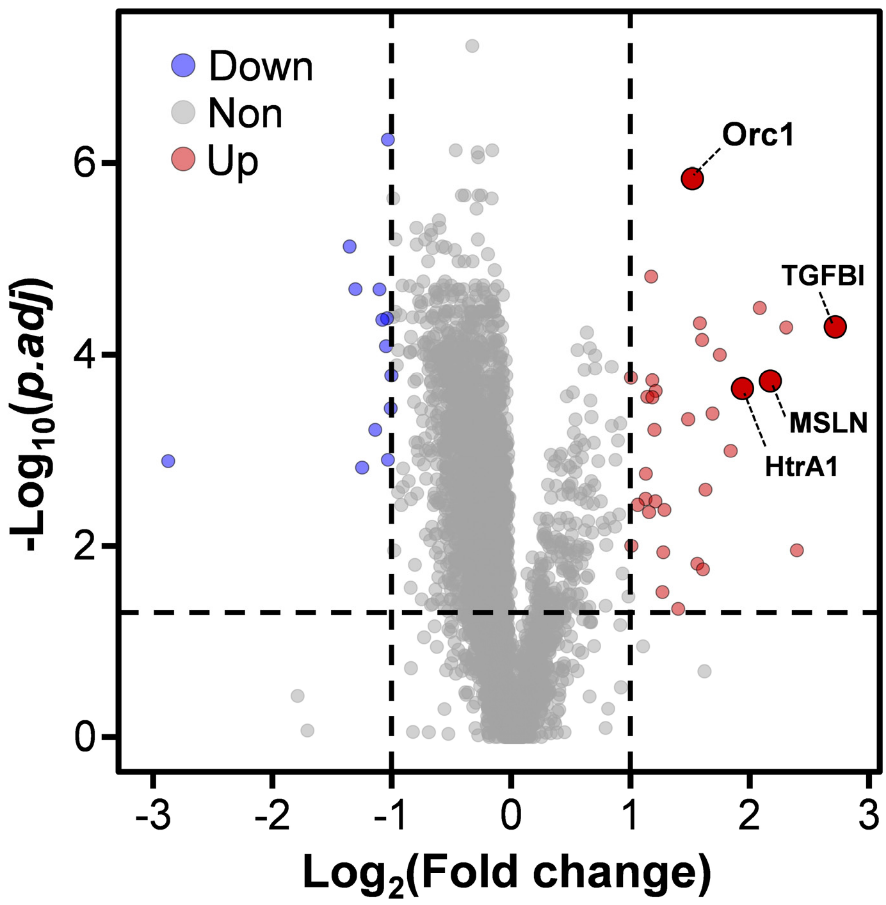

Supplementary Figure 8. Volcano plot showing 33 up-regulated proteins and 13 down-regulated proteins screened out in Cluster 1 compared to Cluster 2 (Wilcoxon test, $p.adj < 0.05$, fold change > 2), including 4 up-regulated proteins (Orc1, TGFBI, MSLN and HtrA1) reported previously to be associated with the migration and invasion of tumor cells (marked with bigger dark red dots). Source data are provided as a Source Data file.

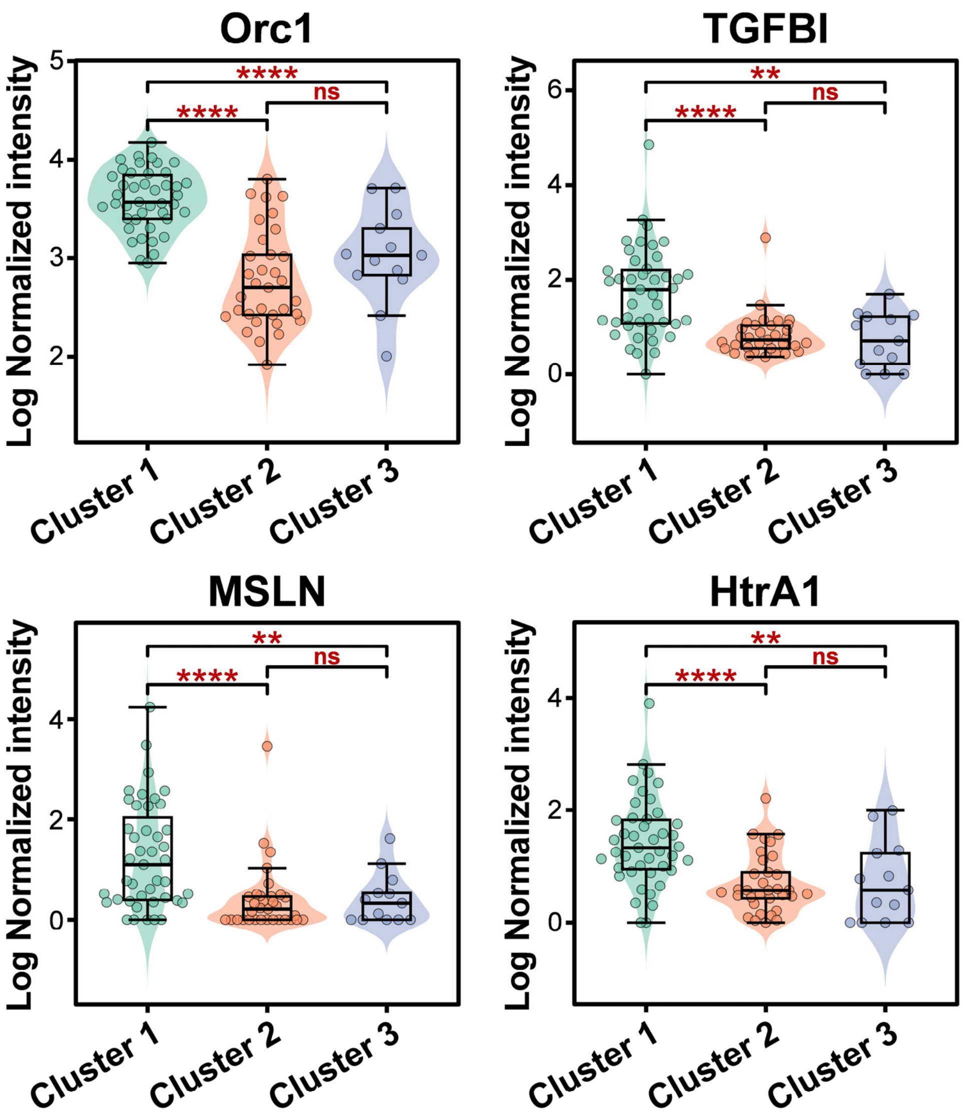

Supplementary Figure 9. Comparisons of the quantitative expression levels of Orc1, TGFBI, MSLN and HtrA1 proteins in cells of cluster 1 (n = 42), cluster 2 (n = 31) and cluster 3 (n = 16). The central lines in the boxes indicate the median values of the corresponding data; the boxes indicate the quartiles; the whiskers extend to a maximum of 1.5 times interquartile range beyond the quartiles; individual data points are overlaid. The differential proteins were determined by fold change > 2 and adjusted p value < 0.05 (two-side Wilcoxon test with Benjamini-Hochberg correction). Significance: **, $0.05 < p \leq 0.01$; ***, $0.01 < p \leq 0.001$; ****, $0.001 < p \leq 0.0001$; ns, $p > 0.05$, no significance. Source data are provided as a Source Data file.

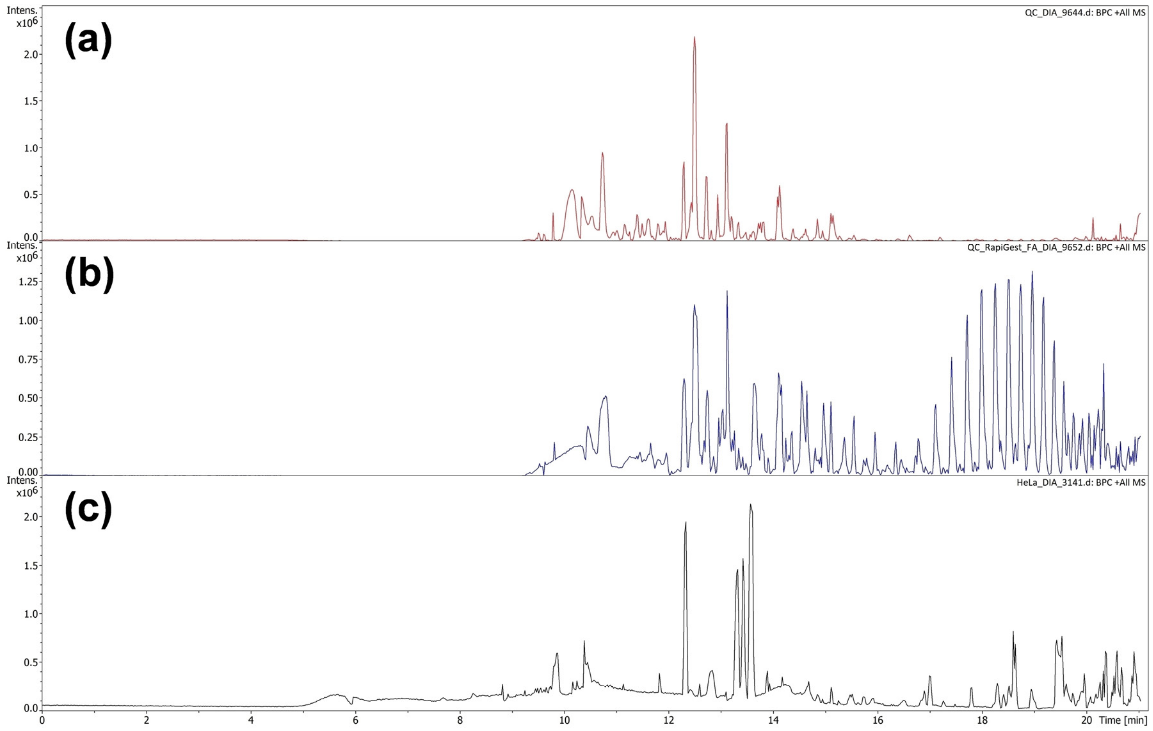

Supplementary Figure 10. Evaluation of the effect of RapiGest SF on single HeLa cell proteomic analysis. (**a**) Base peak chromatogram (BPC) of the QC sample (200 pg of tryptic peptides from HeLa cell digestion, red line); (**b**) BPC of the QC sample doped with excess RapiGest SF and formic acid (100 nL 1.5% (w/v) RapiGest SF, 100 nL formic acid, blue line); (**c**) BPC of a typical single HeLa cell sample (black line). There is no evident peak from RapiGest SF can be observed in the chromatogram of the single HeLa cell sample.

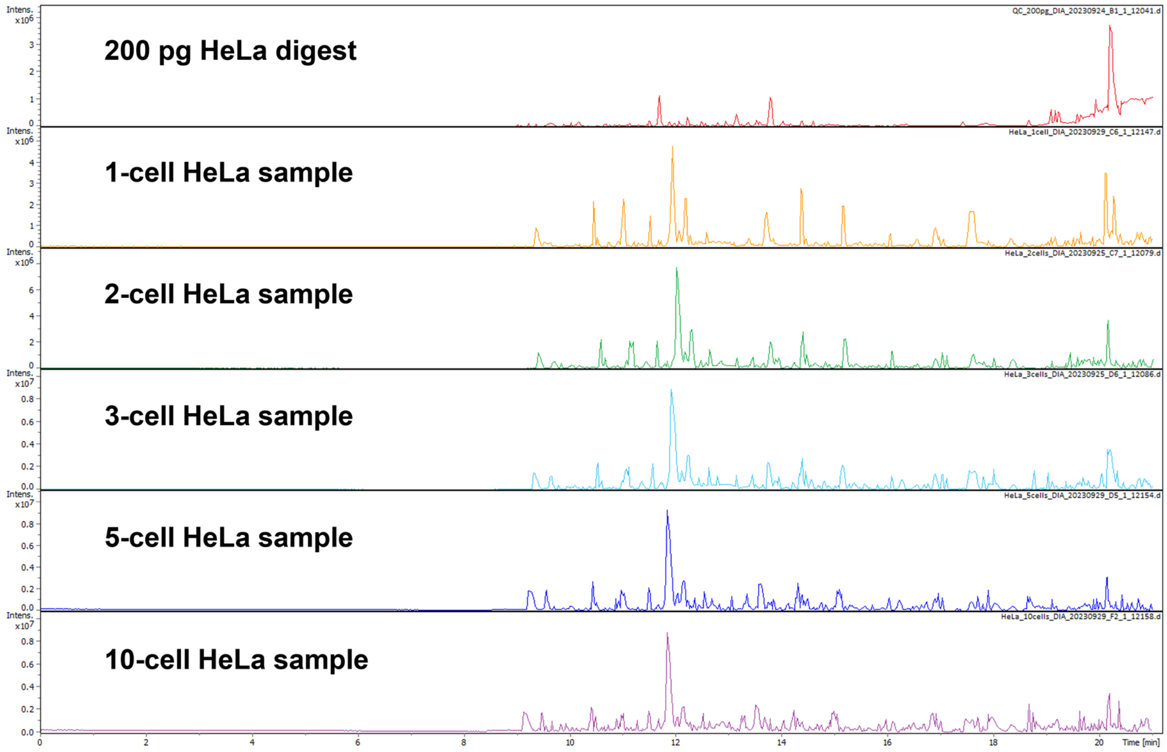

Supplementary Figure 11. Comparison of typical base peak chromatograms (BPC) obtained from the 200 pg HeLa digest, 1-cell HeLa sample, 2-cell HeLa sample, 3-cell HeLa sample, 5-cell HeLa sample, and 10-cell HeLa sample. The peak shapes are generally consistent for the 1-cell, 2-cell, 3-cell, 5-cell, and 10-cell samples, with differences primarily in intensity. However, the peak shapes of the 200 pg HeLa digest differ from the cell samples, with fewer number of peaks and lower peak intensities.

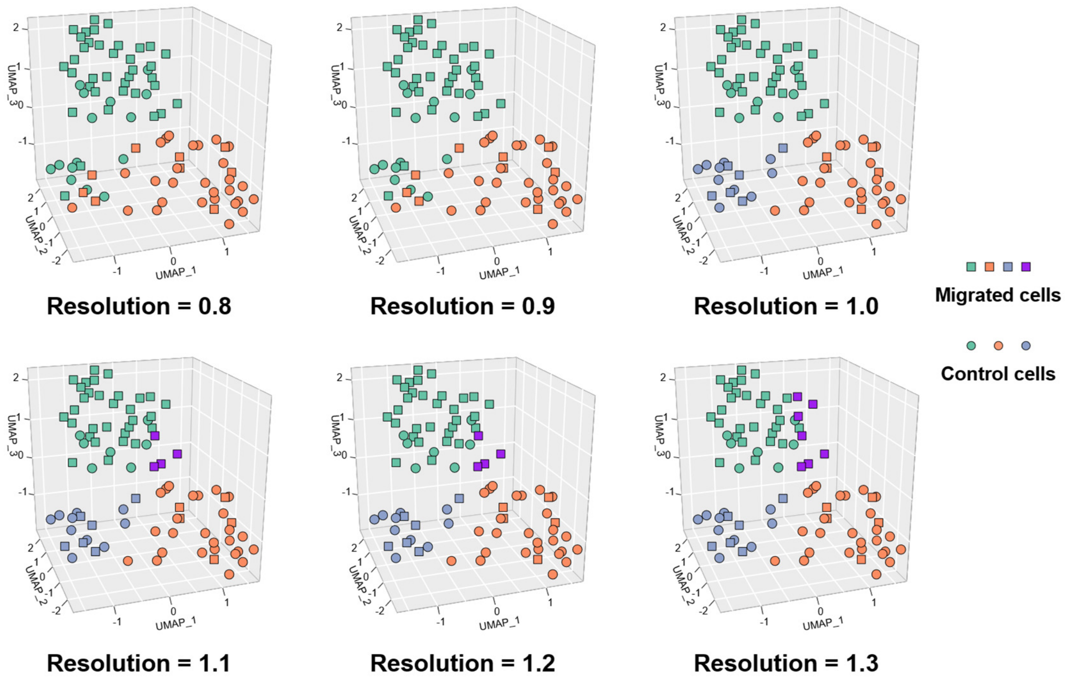

Supplementary Figure 12. Comparison of UMAP clustering results at varying resolutions ranging from 0.8 to 1.3. At resolutions ≤ 0.9, samples coalesce into two clusters with indistinct boundaries. At resolution = 1.0, samples are classified into three distinct and well-defined clusters. At resolutions ≥ 1.1, samples are clustered into more than three clusters, complicating subsequent analyses. Source data are provided as a Source Data file.

Supplementary Note 1. Comparison of UMAP clustering results at varying resolutions. The UMAP clustering in Fig. 5b was performed following the standard workflow. The main parameters affecting the clustering boundary delineation is the resolution. The higher the resolution, the more varieties of clusters are delineated. The results (**Supplementary Figure 12**) show that when the resolution is ≤ 0.9, the samples can only be clustered into 2 clusters, but it is clearly observable that their boundaries are poorly delineated. When the resolution is equal to 1.0, the samples are clearly clustered into 3 well-defined clusters. When resolution ≥ 1.1, the samples were reluctantly divided into 4 clusters, in which the fourth cluster only has four or six samples and its boundaries with the neighboring clusters are unclear, resulting in difficulties in subsequent data analysis. Therefore, resolution of 1.0 with clear and stable clustering results was ultimately chosen for the subsequent analysis.
