# Supplementary material for: Pick-up single-cell proteomic analysis for quantifying up to 3000 proteins in a Mammalian cell
Source: Nat Commun. 2024 Feb 10;15:1279. doi: 10.1038/s41467-024-45659-4 (PMC10858870; doi:10.1038/s41467-024-45659-4)
Supplement: Supplementary file 2 — Description of Additional Supplementary Files [file 41467_2024_45659_MOESM2_ESM.pdf]

Description of Additional Supplementary Files

File Name: Supplementary Data 1
Description: Micrographs showing the target-cells before and after being captured by the PiSPA platform.

File Name: Supplementary Data 2
Description: MS and MS2 spectra of the identified migration-related proteins and peptides.

File Name: Supplementary Data 3
Description: Proteins and unique peptides quantified from migrated cells and control cells in the scratch assay.
