# Supplementary material for: Pick-up single-cell proteomic analysis for quantifying up to 3000 proteins in a Mammalian cell
Source: Nat Commun. 2024 Feb 10;15:1279. doi: 10.1038/s41467-024-45659-4 (PMC10858870; doi:10.1038/s41467-024-45659-4)

Micrographs showing the target-cells before and after being captured by the PiSPA platform.

Single HeLa cell (3099.d)

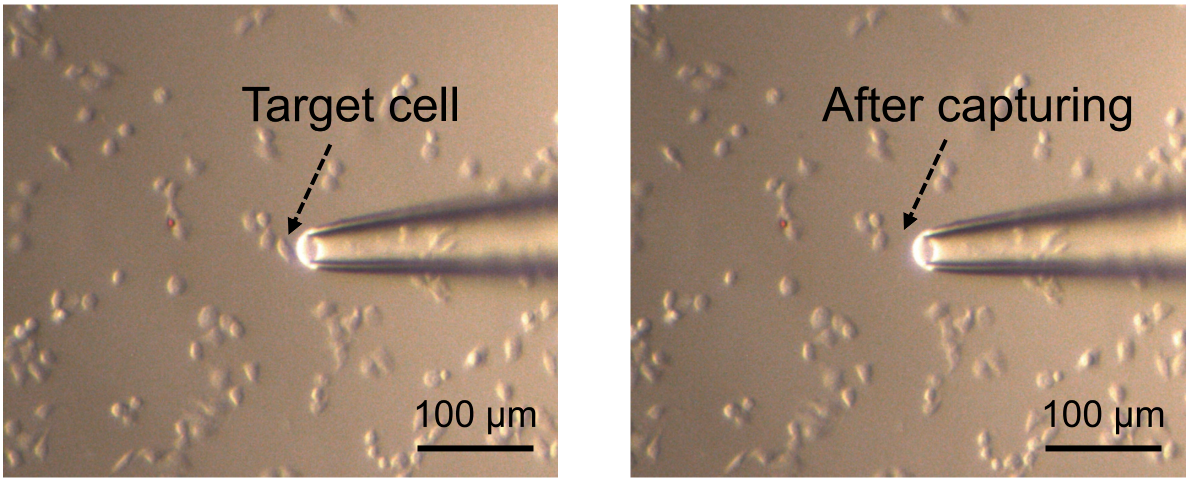

Single HeLa cell (3303.d)

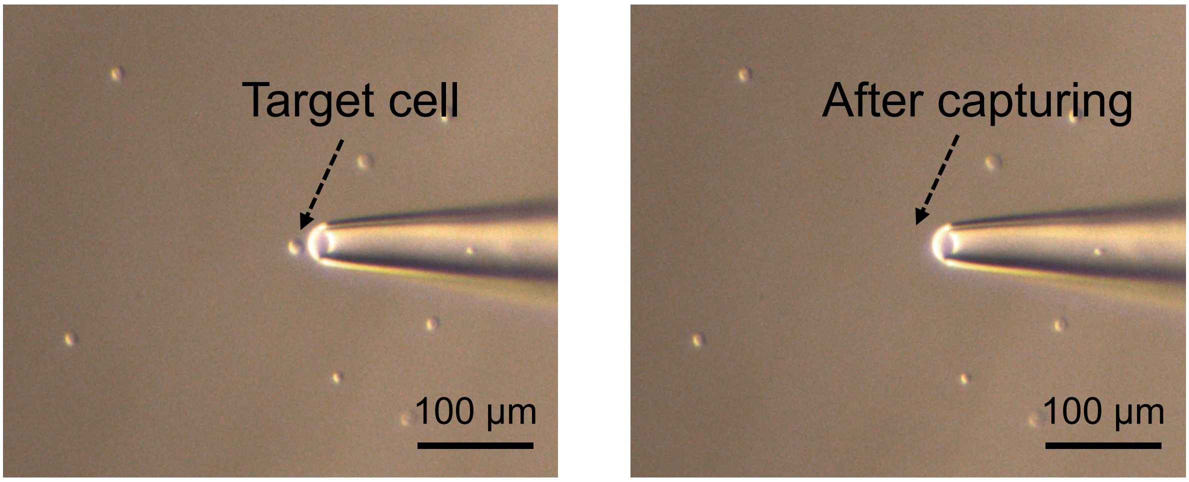

Single HeLa cell (3103.d)

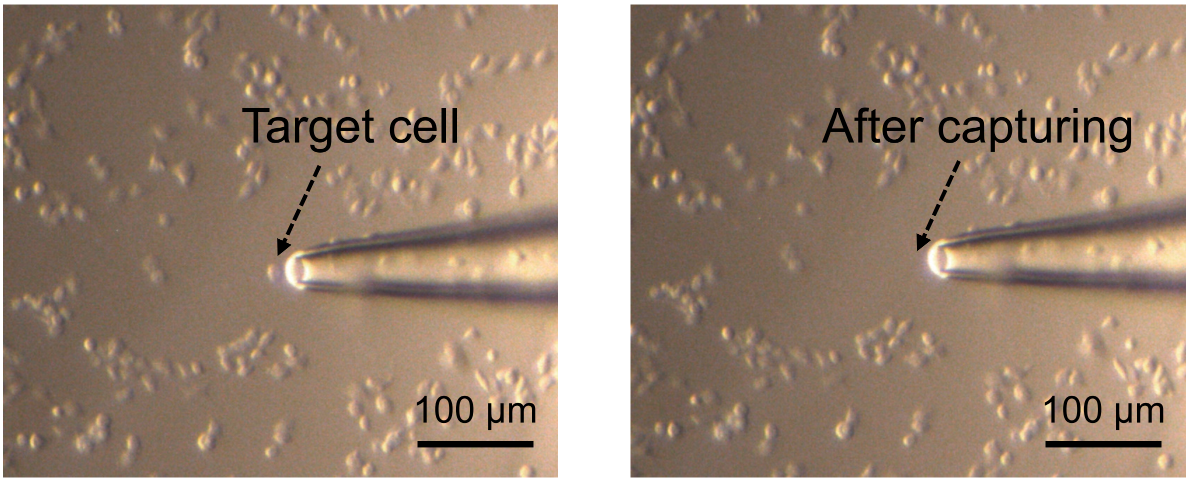

Single HeLa cell (3096.d)

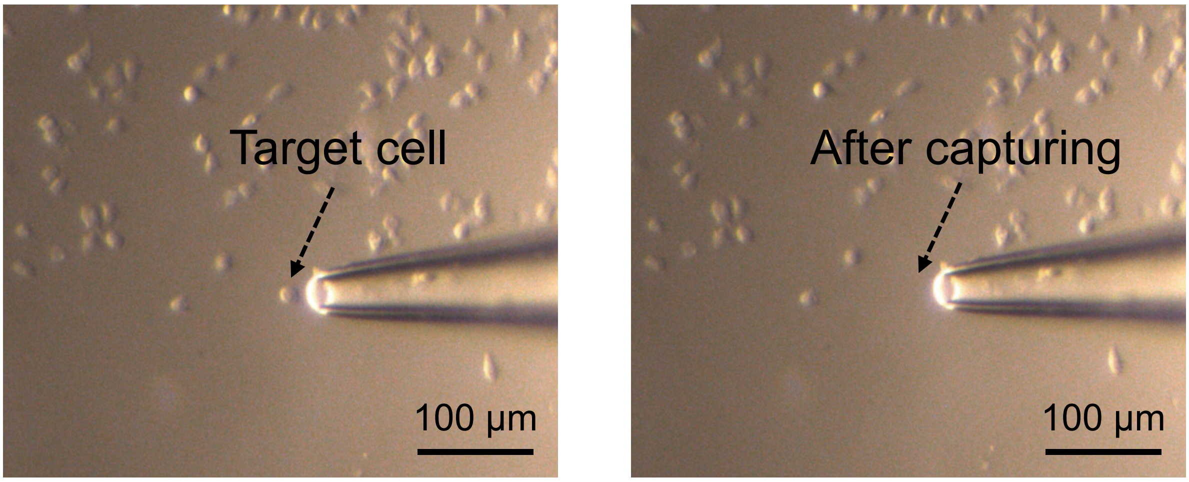

Single HeLa cell (3093.d)

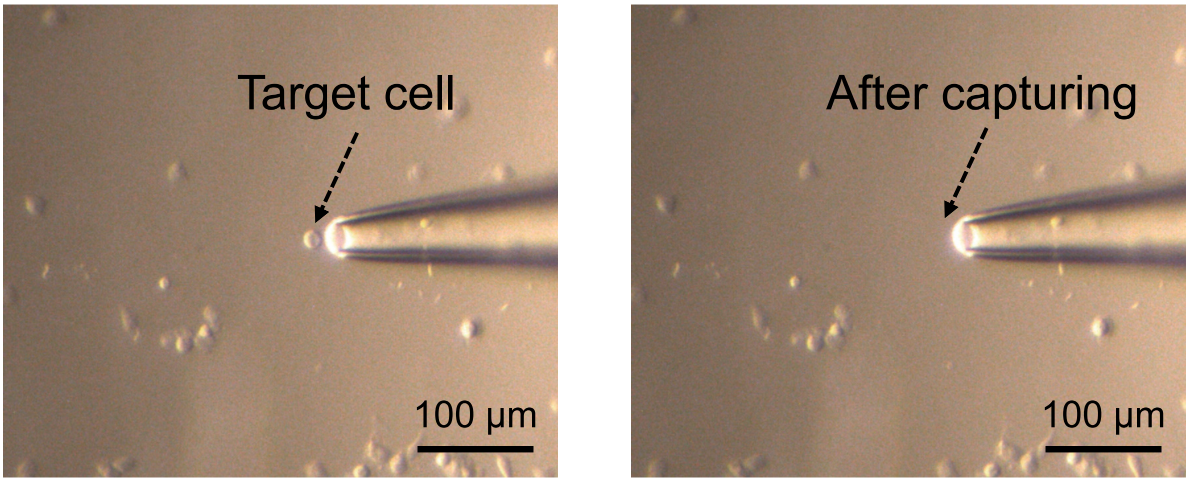

Single HeLa cell (2873.d)

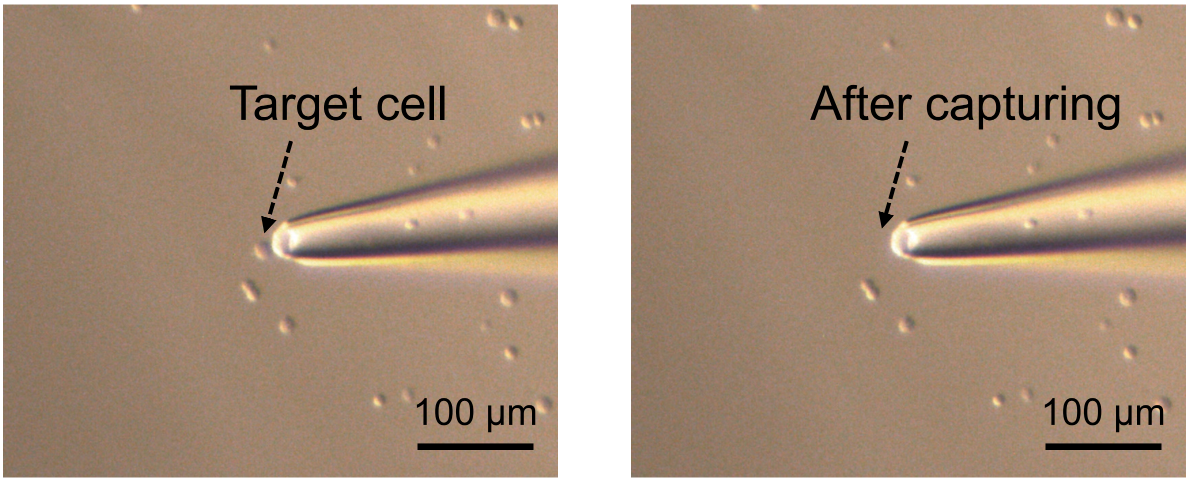

Single HeLa cell (3310.d)

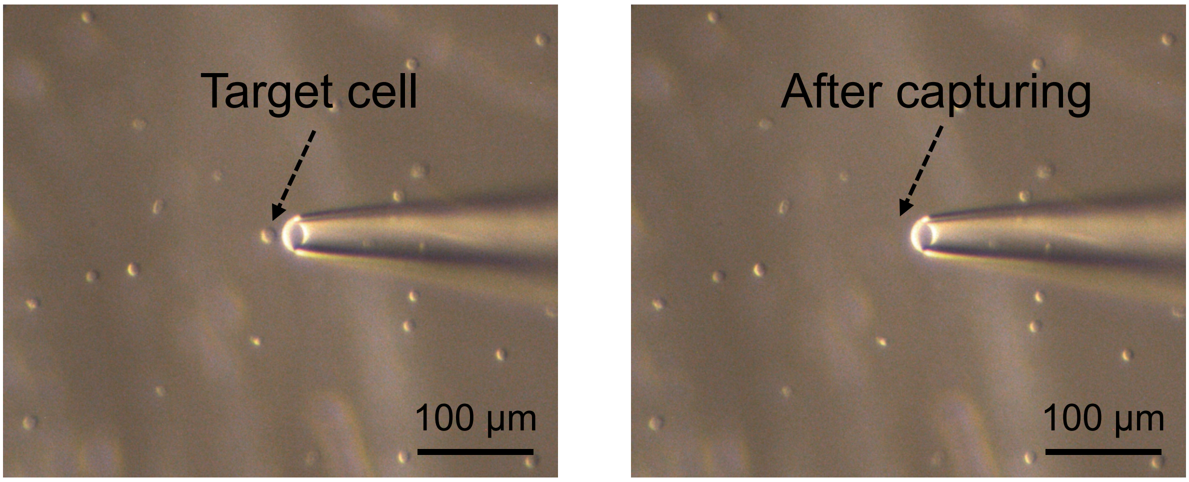

Single HeLa cell (3150.d)

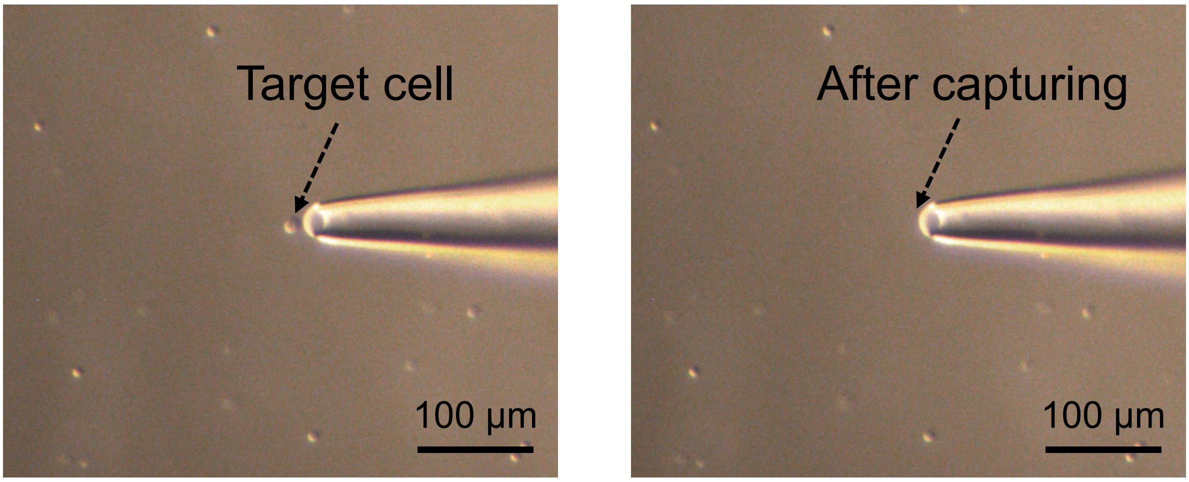

Single HeLa cell (3139.d)

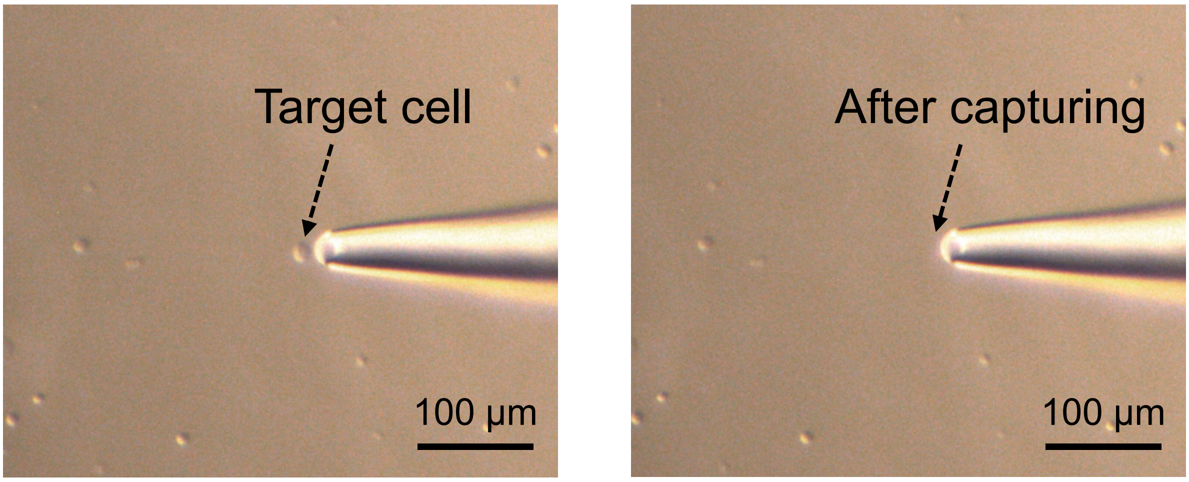

Micrographs showing the target-cell picking-up operation in the scratch assay before and after the capturing of the migrated HeLa cell by the PiSPA platform.

Single HeLa cell (8752.d)

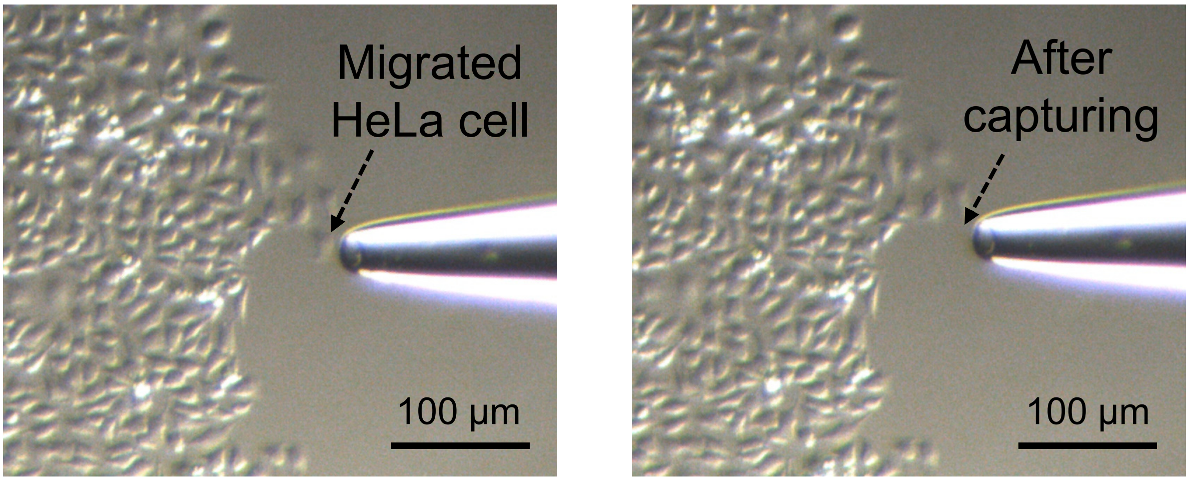

Single HeLa cell (8761.d)

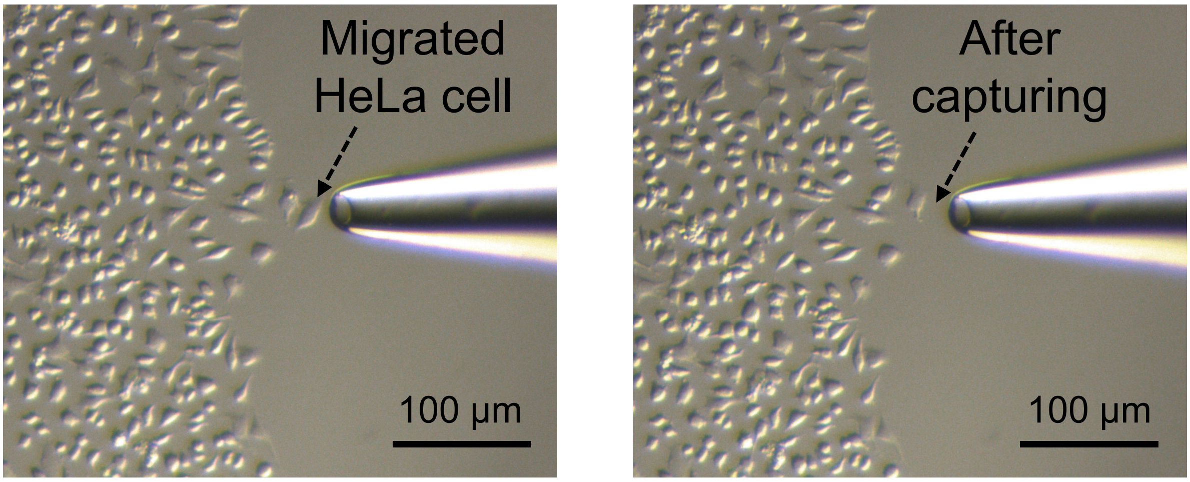

Single HeLa cell (8762.d)

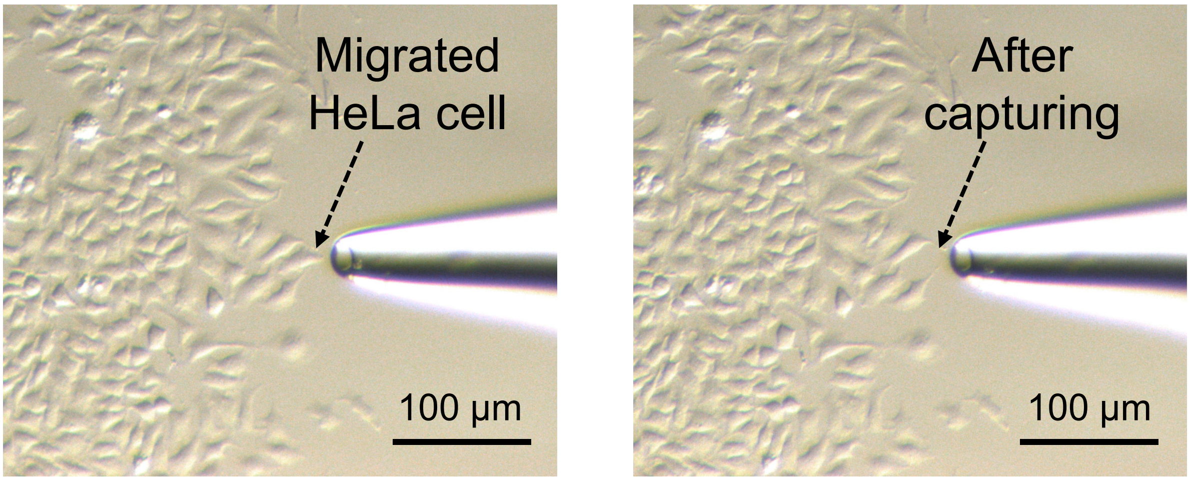

Single HeLa cell (8763.d)

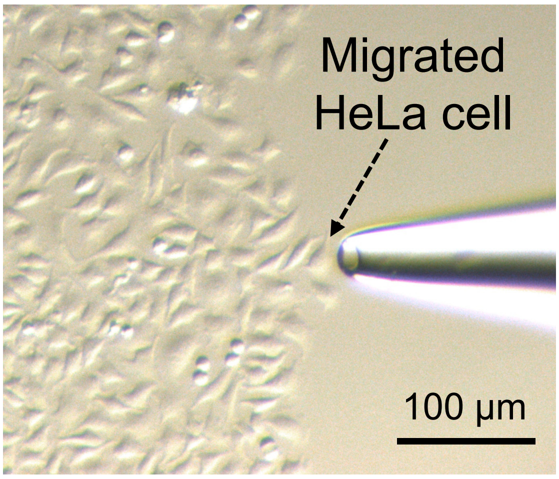

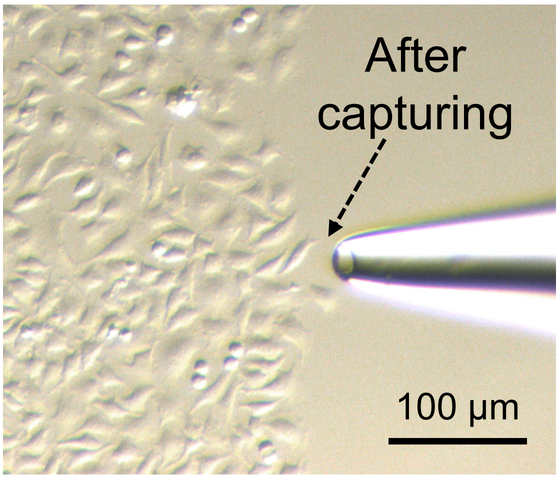

Single HeLa cell (8764.d)

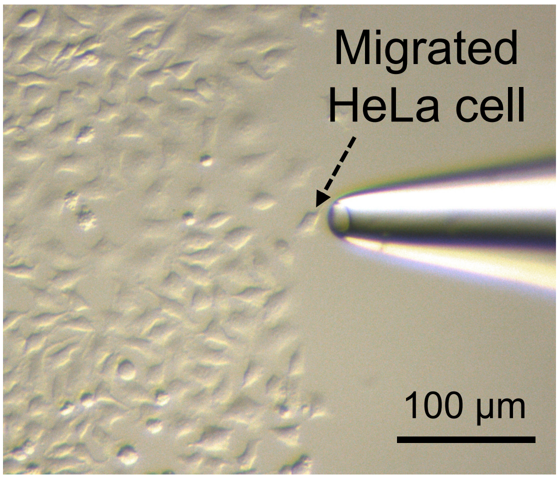

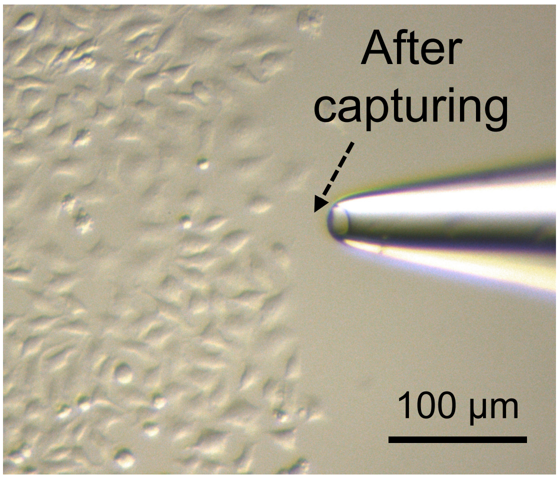

Single HeLa cell (8767.d)

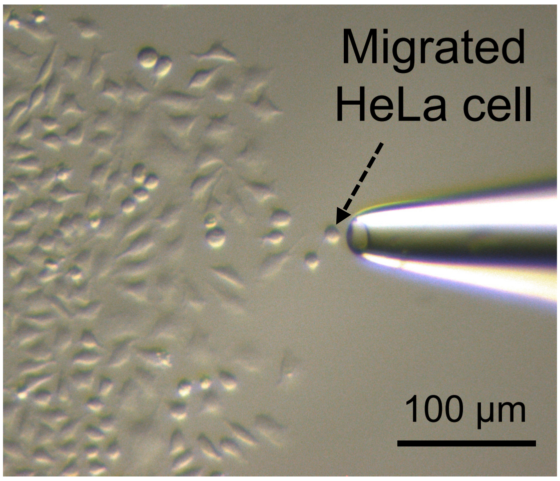

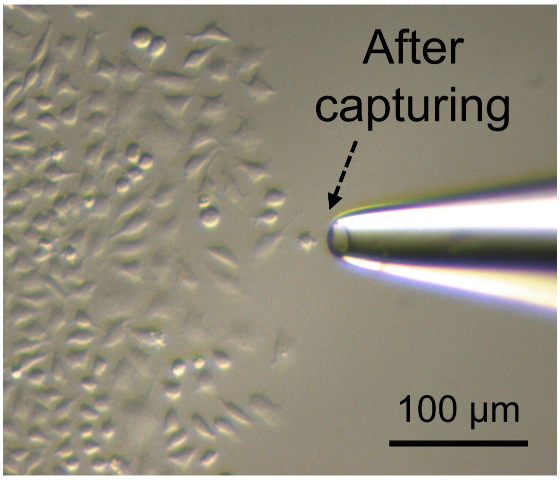

Single HeLa cell (8768.d)

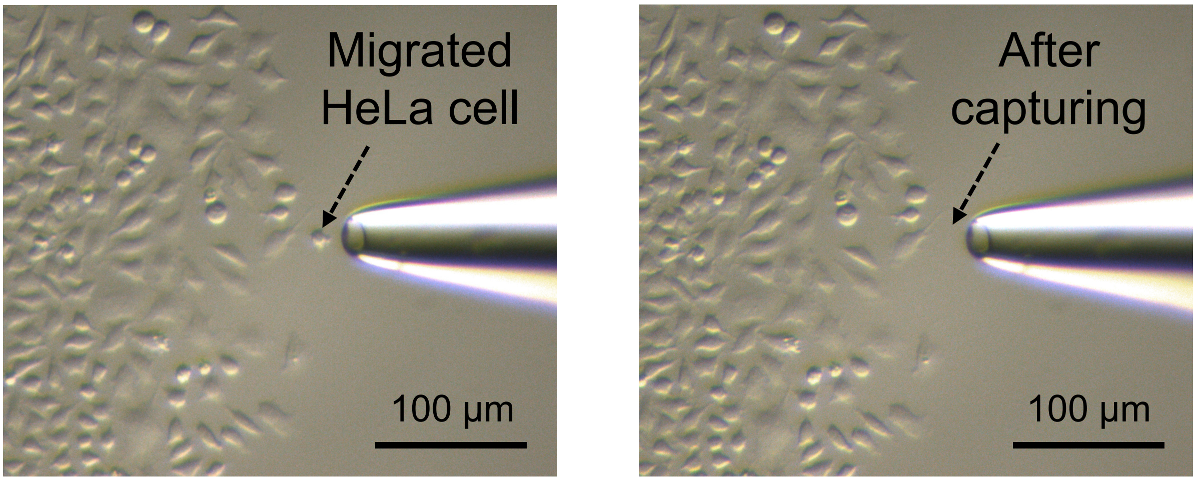

Single HeLa cell (8770.d)

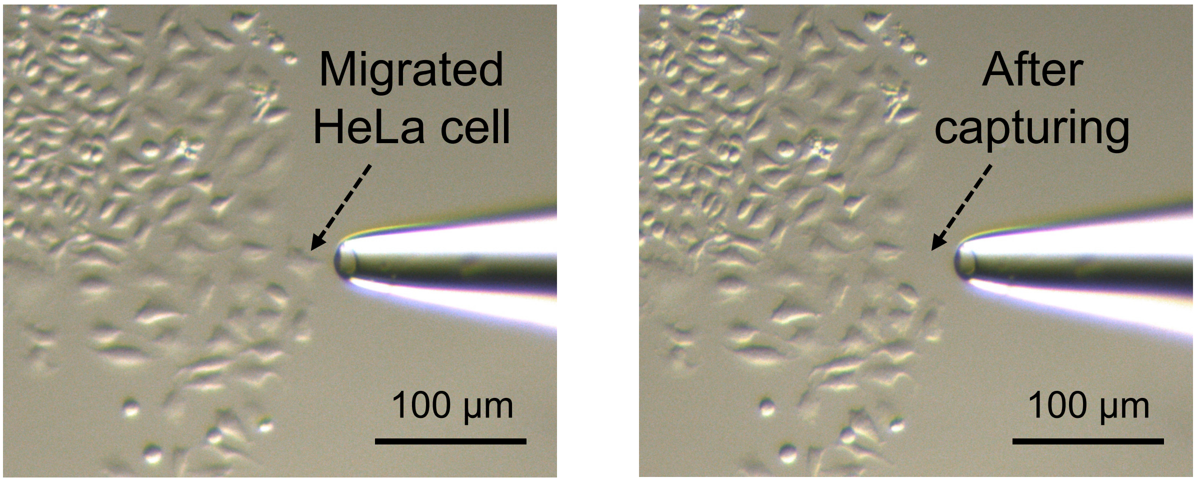

Single HeLa cell (8771.d)

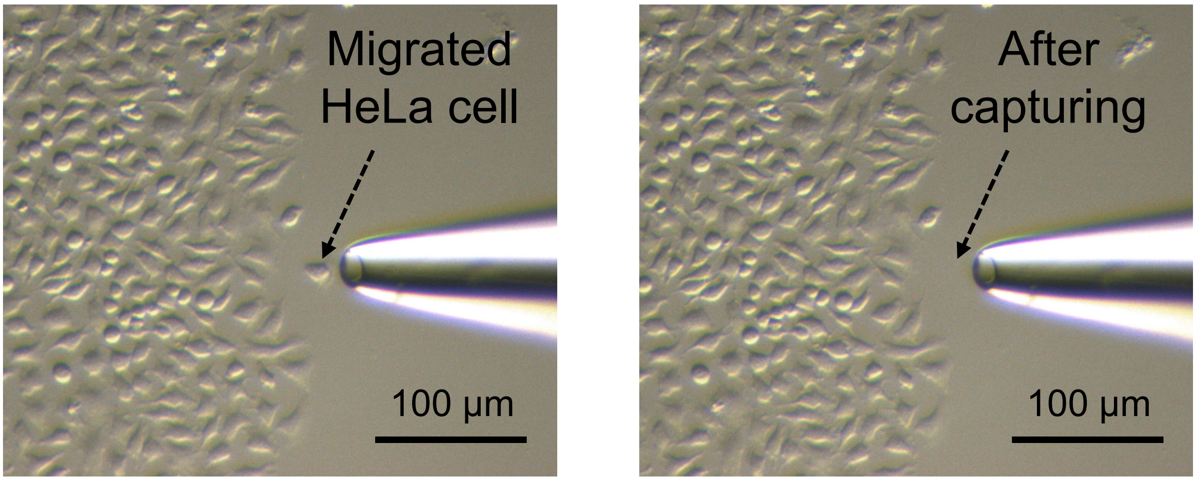

Supplement: Supplementary file 3 — Supplementary Data 1 [file 41467_2024_45659_MOESM3_ESM.pdf]
