# Supplementary material for: Pick-up single-cell proteomic analysis for quantifying up to 3000 proteins in a Mammalian cell
Source: Nat Commun. 2024 Feb 10;15:1279. doi: 10.1038/s41467-024-45659-4 (PMC10858870; doi:10.1038/s41467-024-45659-4)
Supplement: Supplementary file 4 — Supplementary Data 2 [file 41467_2024_45659_MOESM4_ESM.pdf]

CDC42_HUMAN — _NVFDEAILAALEPPEPK_.2

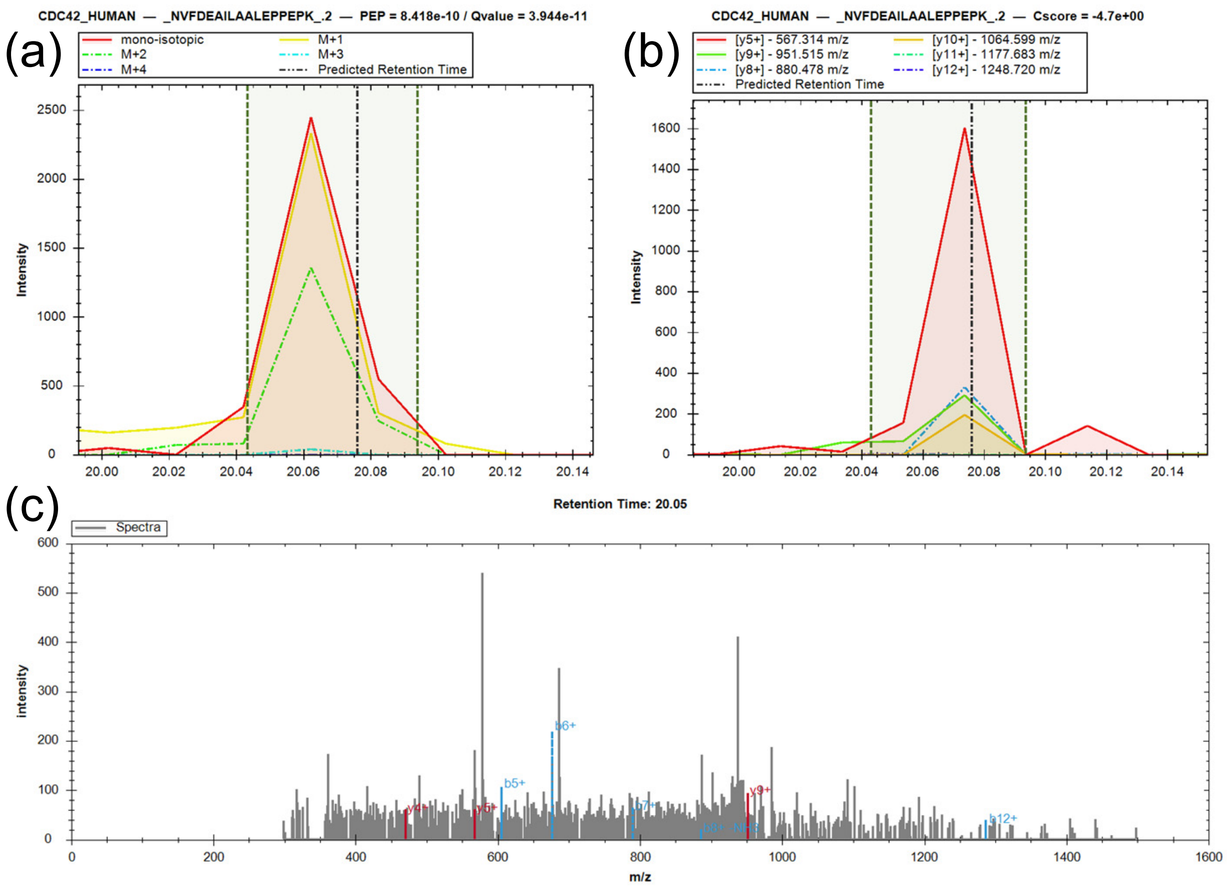

(a) MS1 XIC; (b) MS2 XIC; (c) MS2 Spectrum at Apex

CDC42_HUMAN — _NVFDEAILAALEPPEPK_.3

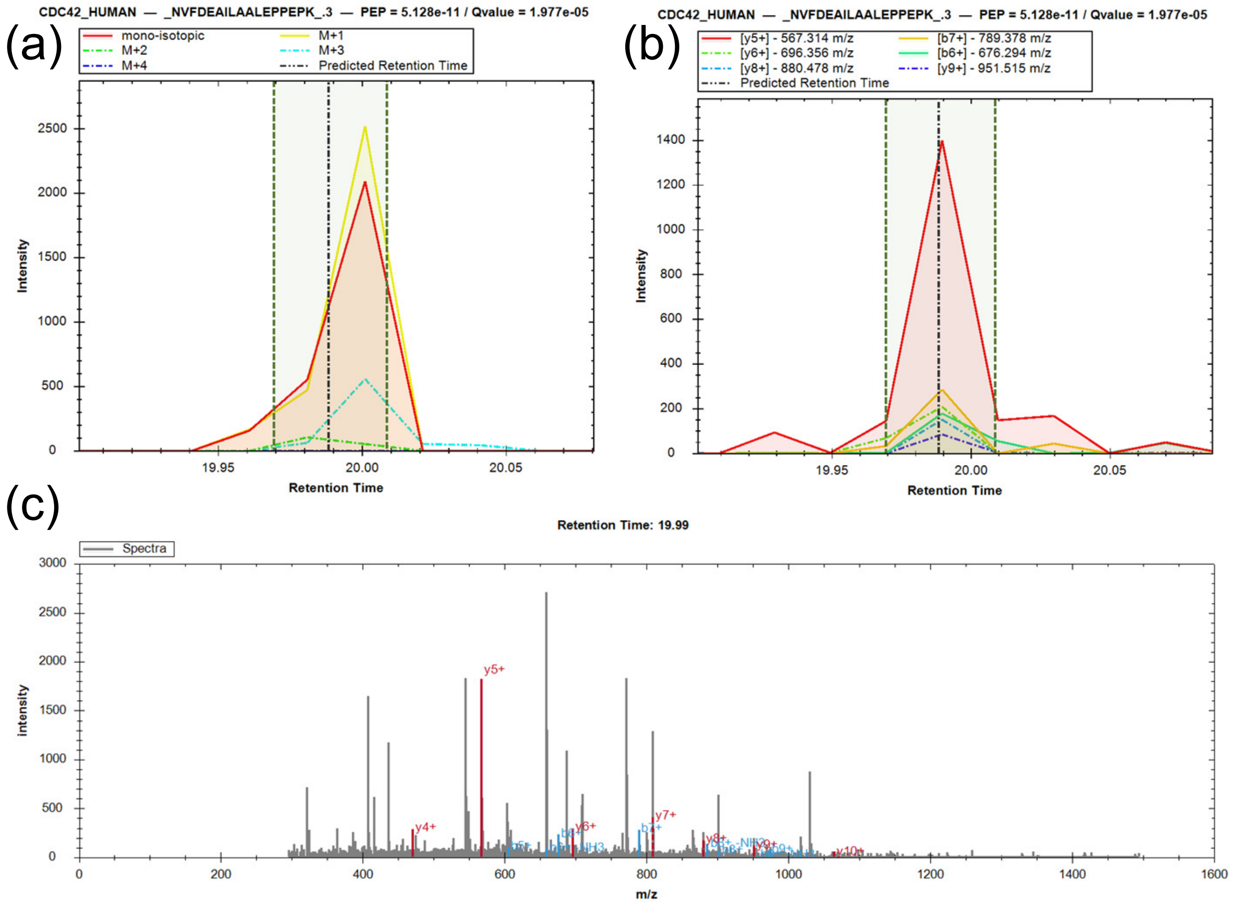

(a) MS1 XIC; (b) MS2 XIC; (c) MS2 Spectrum at Apex

CDC42_HUMAN — _QKPITPETAEK_.2

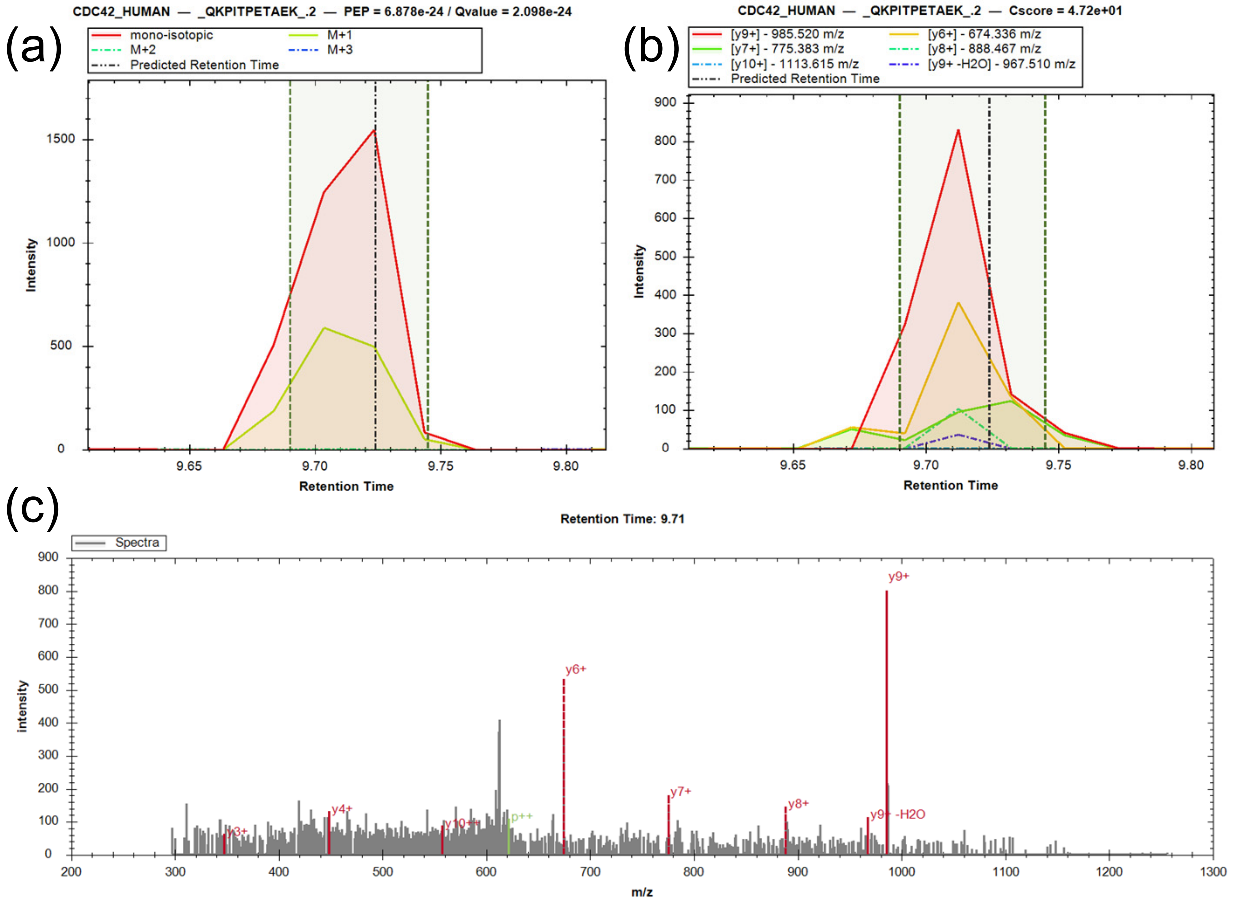

(a) MS1 XIC; (b) MS2 XIC; (c) MS2 Spectrum at Apex

CDC42_HUMAN — _QKPITPETAEK_.3

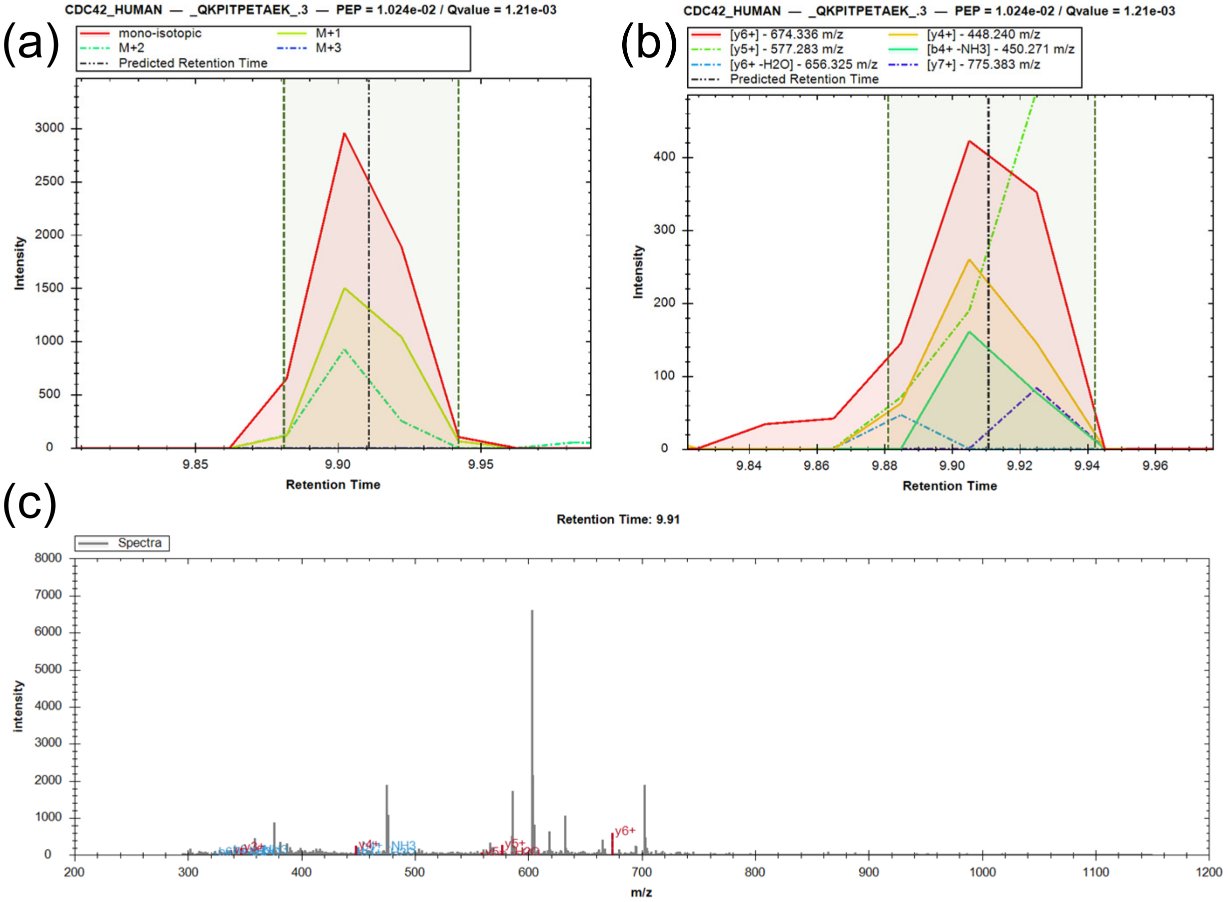

(a) MS1 XIC; (b) MS2 XIC; (c) MS2 Spectrum at Apex

CDC42_HUMAN — _TPFLLVGTQIDLRDDPSTIEK_.3

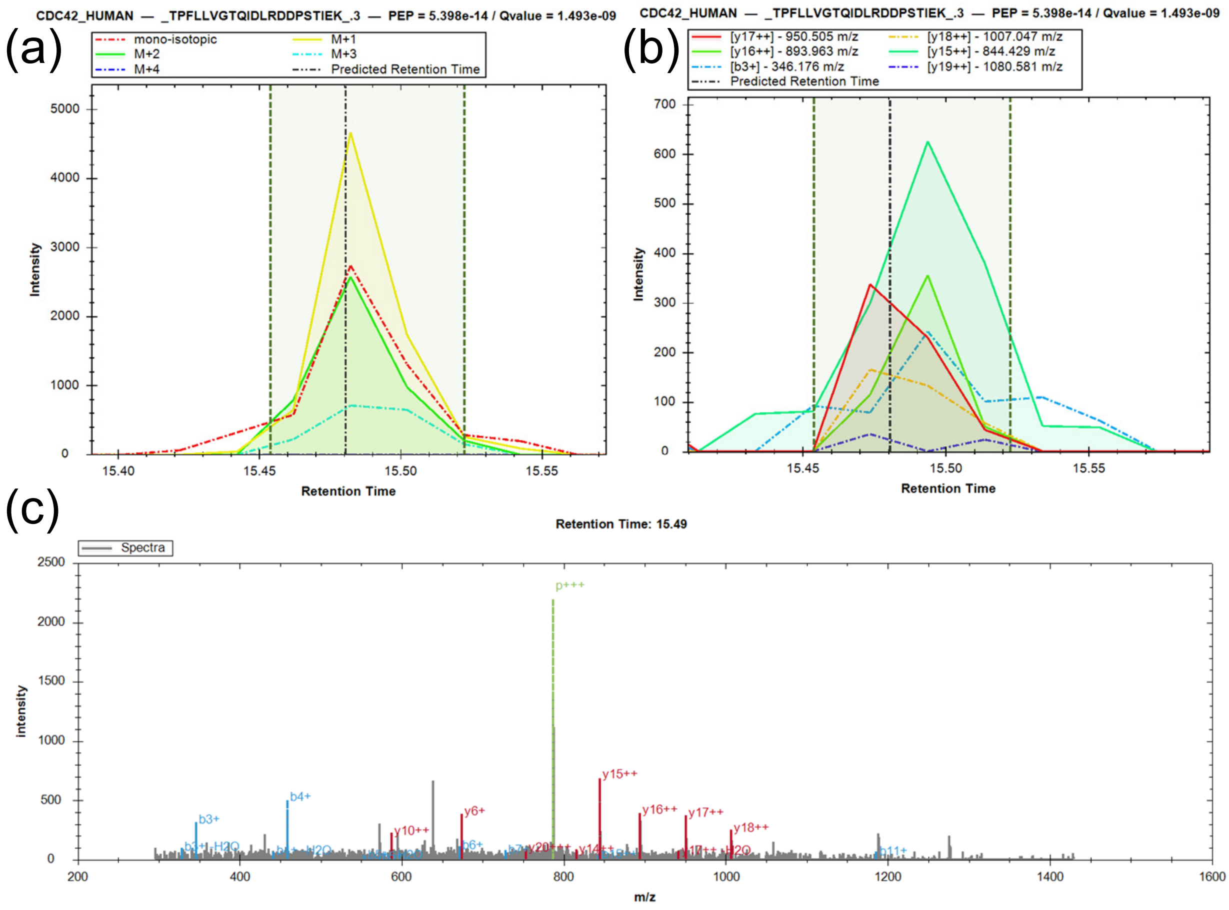

(a) MS1 XIC; (b) MS2 XIC; (c) MS2 Spectrum at Apex

RHOA_HUMAN — _LVIVGDGAC[Carbamidomethyl (C)]GK_.2

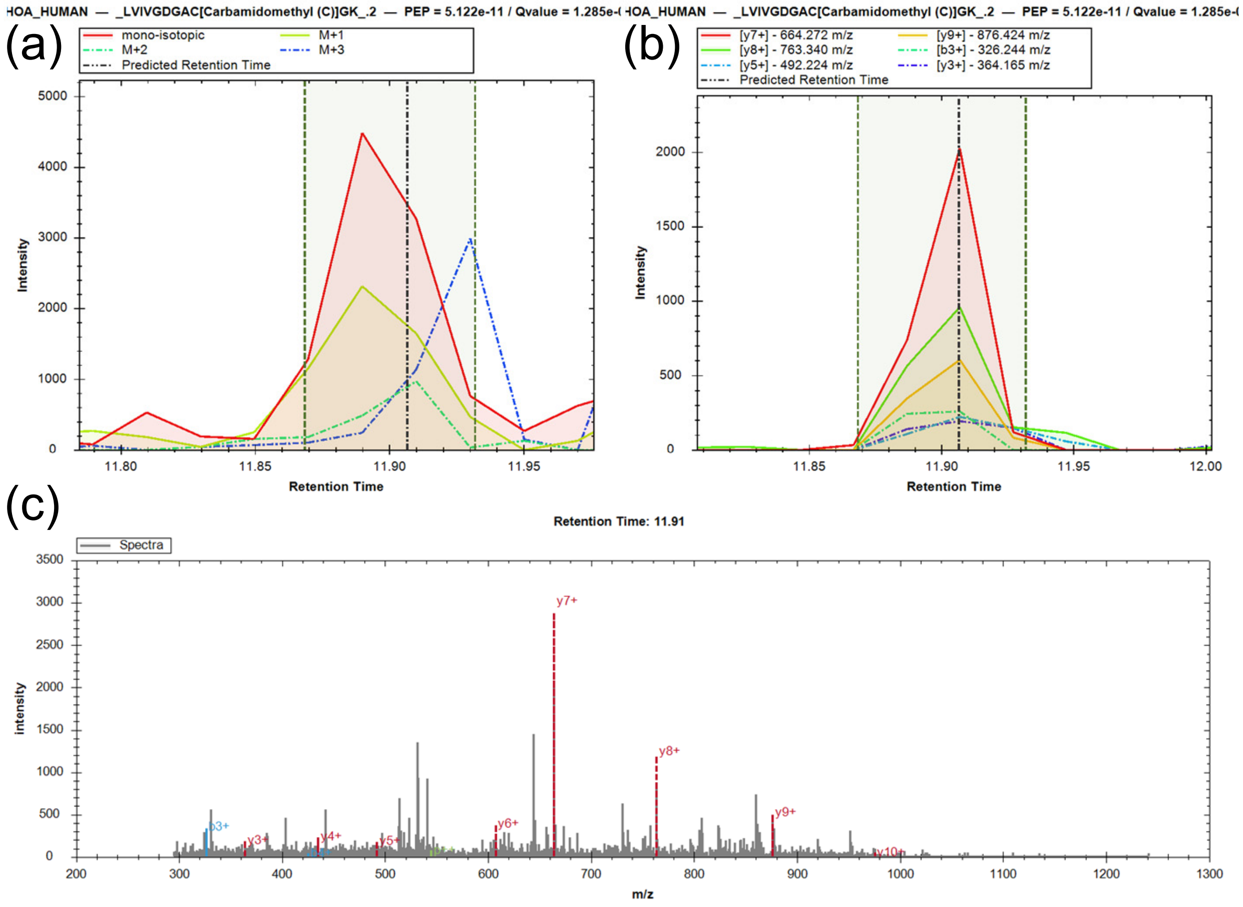

(a) MS1 XIC; (b) MS2 XIC; (c) MS2 Spectrum at Apex

RHOA_HUMAN — _QEPVKPEEGR_.2

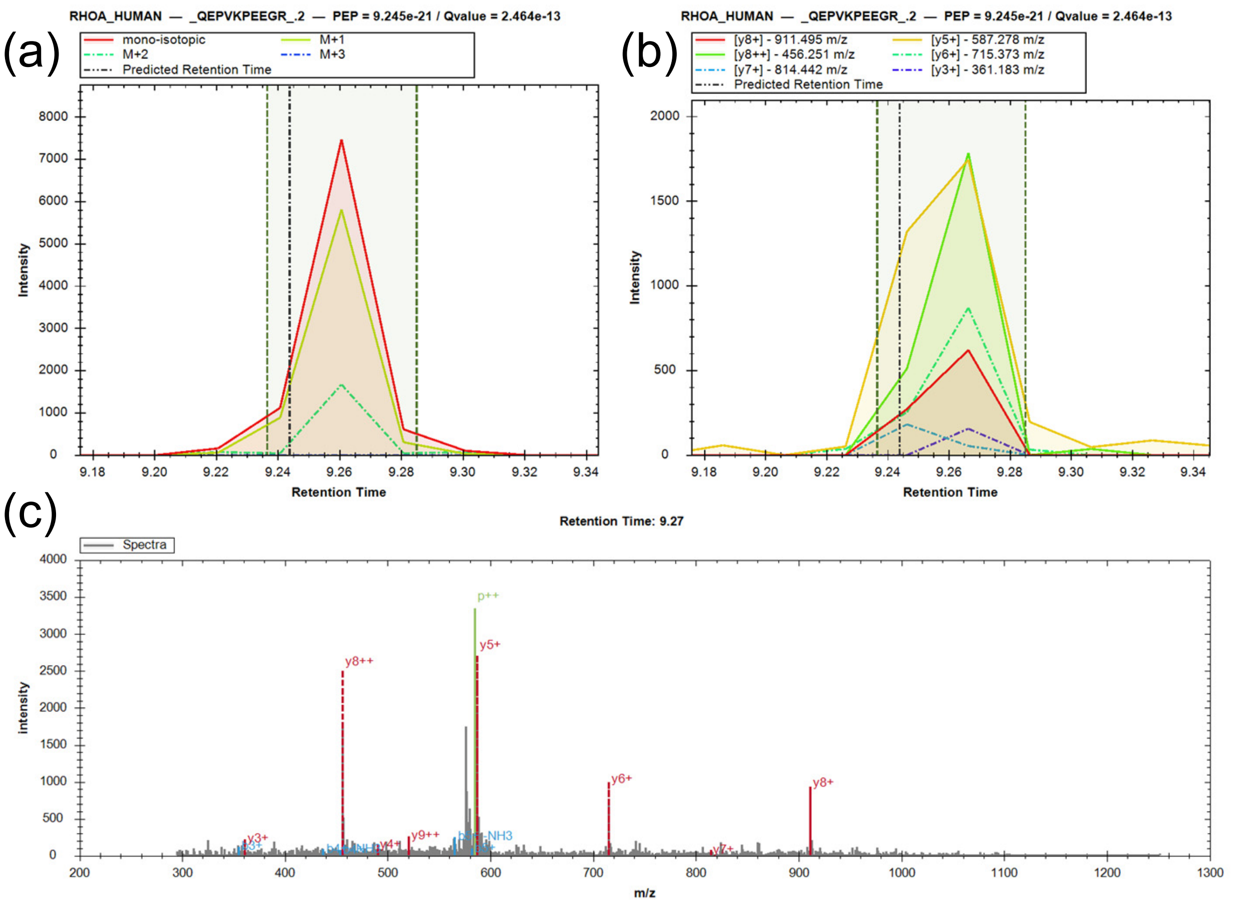

(a) MS1 XIC; (b) MS2 XIC; (c) MS2 Spectrum at Apex

RHOA_HUMAN — _QEPVKPEEGRDM[Oxidation (M)]ANR_.3

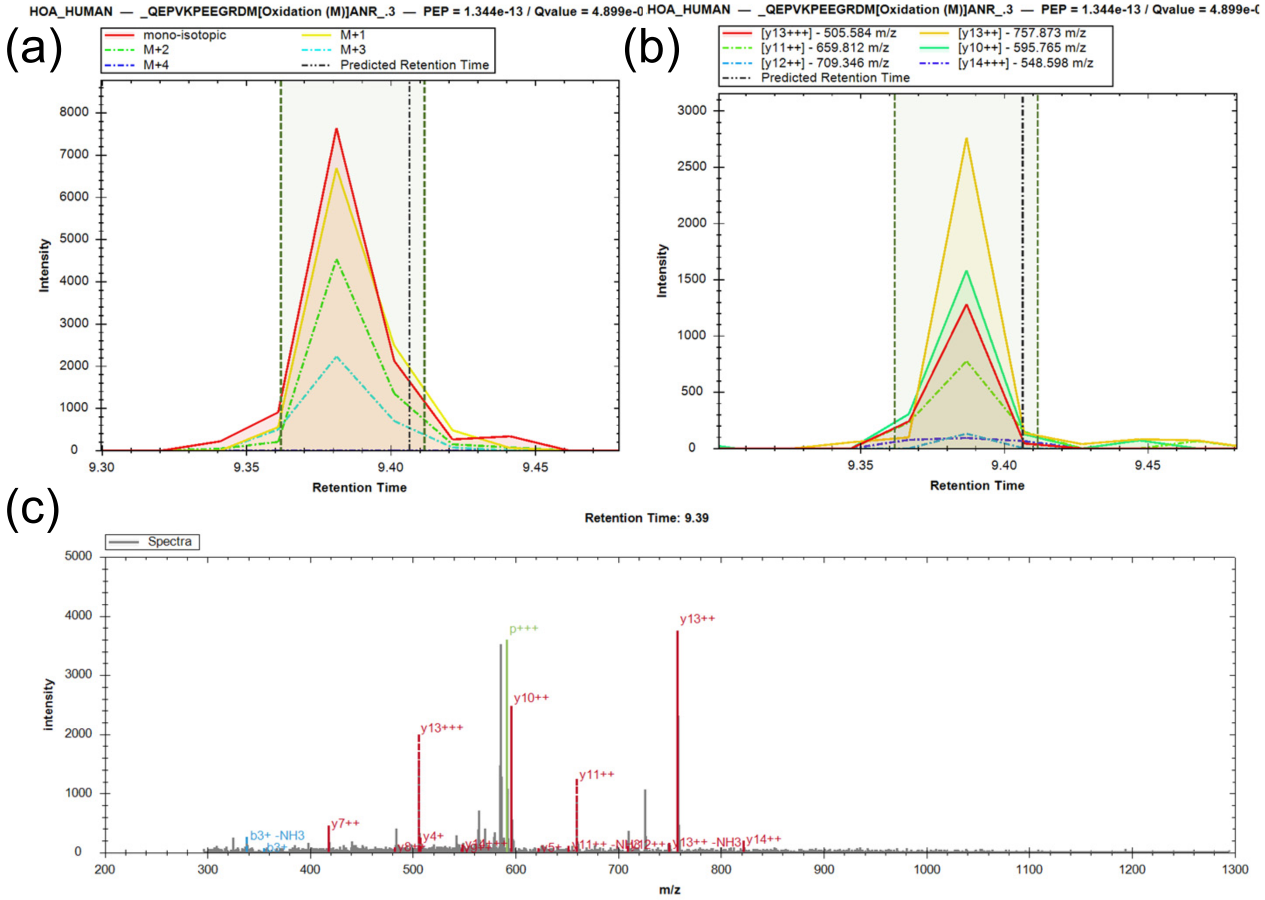

(a) MS1 XIC; (b) MS2 XIC; (c) MS2 Spectrum at Apex

RAC1_HUMAN — _LTPITYPQGLAM[Oxidation (M)]AK_.2

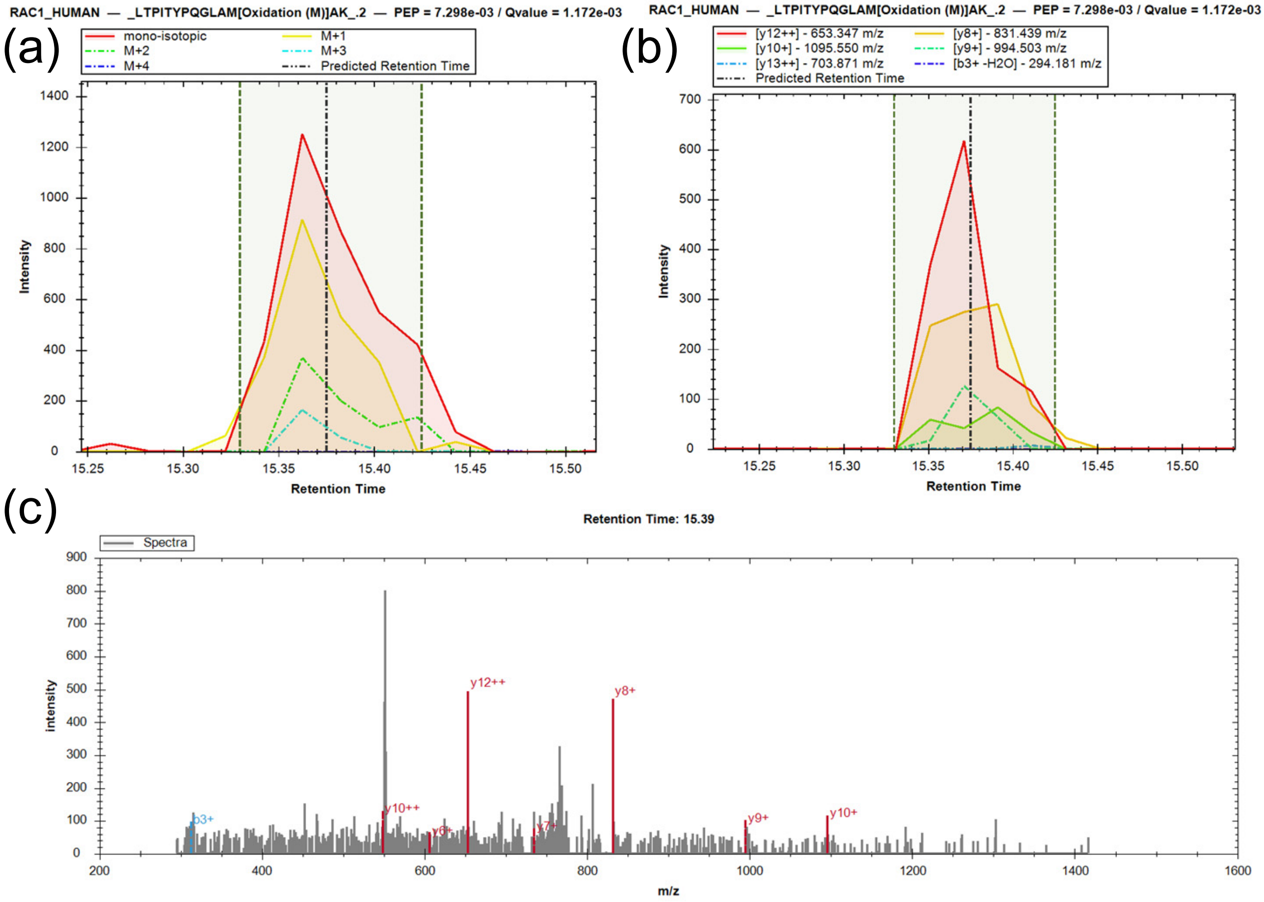

(a) MS1 XIC; (b) MS2 XIC; (c) MS2 Spectrum at Apex

RAC1_HUMAN — _TVFDEAIR_.2

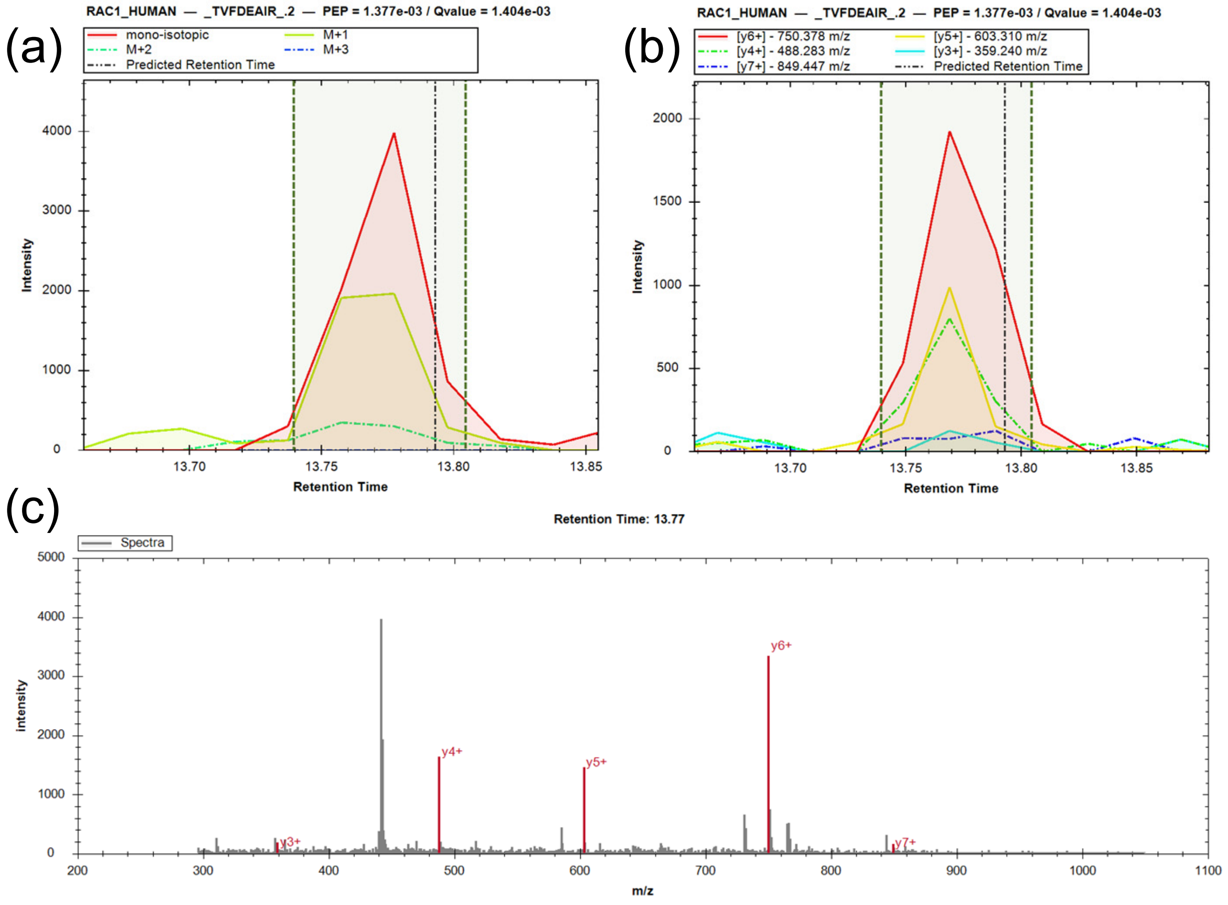

(a) MS1 XIC; (b) MS2 XIC; (c) MS2 Spectrum at Apex

RAC1_HUMAN — _YLEC[Carbamidomethyl (C)]SALTQR_.2

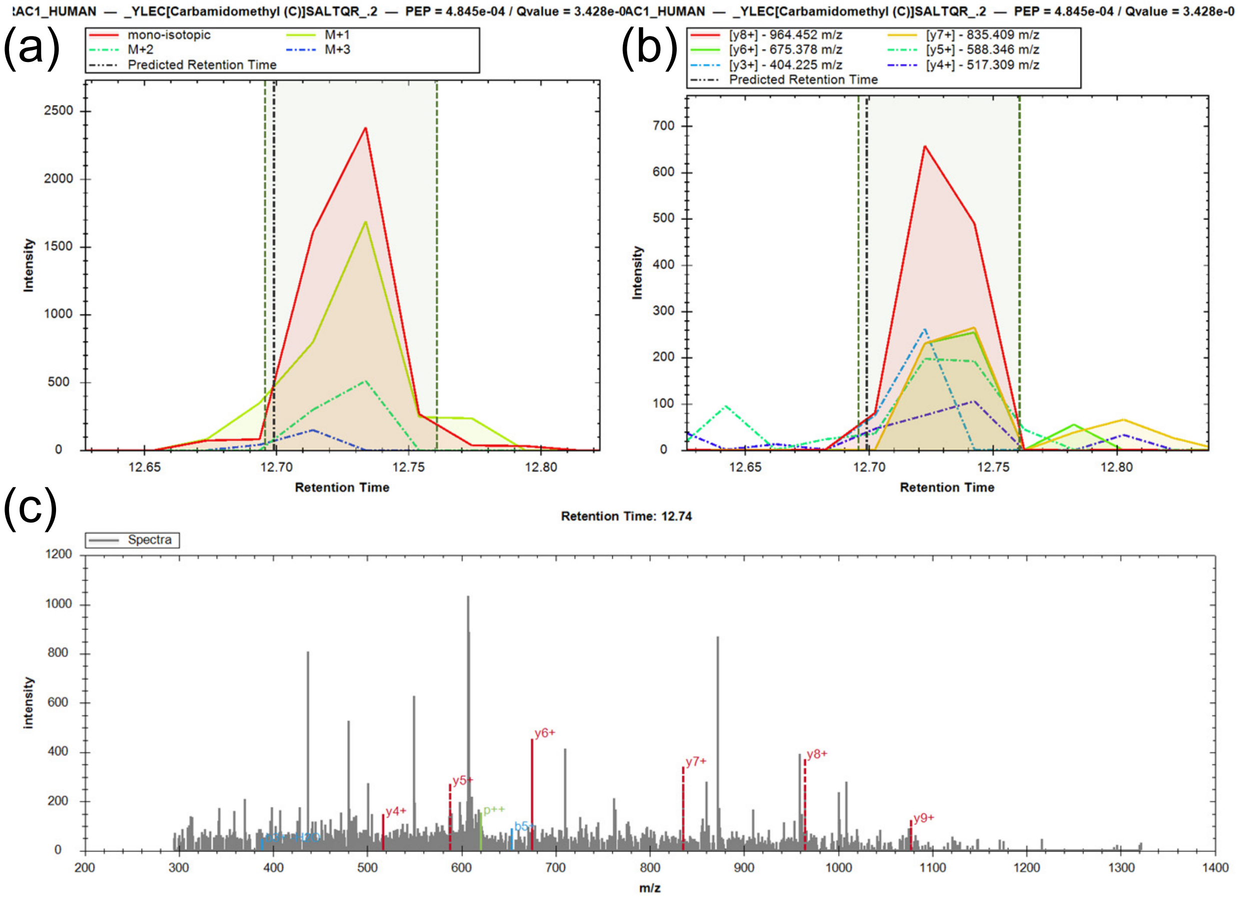

(a) MS1 XIC; (b) MS2 XIC; (c) MS2 Spectrum at Apex
