# Supplementary material for: Pick-up single-cell proteomic analysis for quantifying up to 3000 proteins in a Mammalian cell
Source: Nat Commun. 2024 Feb 10;15:1279. doi: 10.1038/s41467-024-45659-4 (PMC10858870; doi:10.1038/s41467-024-45659-4)
Supplement: Supplementary file 6 — Reporting Summary [file 41467_2024_45659_MOESM6_ESM.pdf]

nature portfolio

Corresponding author(s): Qun Fang

Last updated by author(s): Dec 18, 2023

Reporting Summary

Nature Portfolio wishes to improve the reproducibility of the work that we publish. This form provides structure for consistency and transparency in reporting. For further information on Nature Portfolio policies, see our Editorial Policies and the Editorial Policy Checklist.

Statistics

For all statistical analyses, confirm that the following items are present in the figure legend, table legend, main text, or Methods section.

n/a	Confirmed	
☐	☒	The exact sample size (*n*) for each experimental group/condition, given as a discrete number and unit of measurement
☐	☒	A statement on whether measurements were taken from distinct samples or whether the same sample was measured repeatedly
☐	☒	The statistical test(s) used AND whether they are one- or two-sided<br>*Only common tests should be described solely by name; describe more complex techniques in the Methods section.*
☒	☐	A description of all covariates tested
☒	☐	A description of any assumptions or corrections, such as tests of normality and adjustment for multiple comparisons
☐	☒	A full description of the statistical parameters including central tendency (e.g. means) or other basic estimates (e.g. regression coefficient) AND variation (e.g. standard deviation) or associated estimates of uncertainty (e.g. confidence intervals)
☐	☒	For null hypothesis testing, the test statistic (e.g. *F*, *t*, *r*) with confidence intervals, effect sizes, degrees of freedom and *P* value noted<br>*Give P values as exact values whenever suitable.*
☒	☐	For Bayesian analysis, information on the choice of priors and Markov chain Monte Carlo settings
☒	☐	For hierarchical and complex designs, identification of the appropriate level for tests and full reporting of outcomes
☐	☒	Estimates of effect sizes (e.g. Cohen's *d*, Pearson's *r*), indicating how they were calculated

Our web collection on statistics for biologists contains articles on many of the points above.

Software and code

Policy information about availability of computer code

Data collection	Mass spectrometry data was collected using Compass Hystar software (version 5.1).
Data analysis	The DDA raw files were analyzed with SpectroMine software (version3.2, Biognosys AG, Schlieren, Switzerland). The DIA raw files were analyzed with DIA-NN software (version 1.8).<br>The pairwise correlation analysis between the protein quantification data of the QC samples were performed by Python (version 3.9.15) and the package Pandas (version 1.4.4). The fold change in protein abundance and density distribution of coefficient of variation were analyzed by R (version 4.1.3).<br>Single-cell proteomics data were analyzed by R (version 4.1.3) and the R package Seurat (version 4.3.0). Batch effects of samples were corrected by the R package harmony (version 0.1.1). Kyoto Encyclopedia of Genes and Genomes (KEGG) enrichment was carried out by the R package clusterProfiler (version 4.8.1).<br>The 2D-UMAP of the tumor cells was visualized by the Python package umap-learn (version 0.5.3). The 3D-UMAP in the cell migration study was visualized by the R package plot3D (version 1.4), and the heatmap was visualized by the R package ComplexHeatmap (version 2.16.0). Other data were visualized by the R package ggplot2 (version 3.3.5).

For manuscripts utilizing custom algorithms or software that are central to the research but not yet described in published literature, software must be made available to editors and reviewers. We strongly encourage code deposition in a community repository (e.g. GitHub). See the Nature Portfolio guidelines for submitting code & software for further information.

Data

Policy information about availability of data

All manuscripts must include a data availability statement. This statement should provide the following information, where applicable:

- Accession codes, unique identifiers, or web links for publicly available datasets
- A description of any restrictions on data availability
- For clinical datasets or third party data, please ensure that the statement adheres to our policy

The mass spectrometry proteomics data generated in this study have been deposited to the ProteomeXchange Consortium via the iProX partner repository with the dataset identifier PXD041966 or IPX0006351000 [https://www.iprox.cn/page/project.html?id=IPX0006351000]. Homo sapiens (accession: UP000005640, taxon ID: 9606, 20,626 entries, access date 2021-01), Escherichia coli (strain K12) (taxon ID: 83333, 4530 entries, access date 2023-07) and Saccharomyces cerevisiae (strain ATCC 204508 / S288c) (taxon ID: 559292, 6727 entries, access date 2023-07) protein databases were downloaded from UniProt [https://www.uniprot.org], and have also been deposited to the ProteomeXchange/iProX repository. Source data are provided with this paper.

Research involving human participants, their data, or biological material

Policy information about studies with human participants or human data. See also policy information about sex, gender (identity/presentation), and sexual orientation and race, ethnicity and racism.

Reporting on sex and gender	N/A
Reporting on race, ethnicity, or other socially relevant groupings	N/A
Population characteristics	N/A
Recruitment	N/A
Ethics oversight	N/A

Note that full information on the approval of the study protocol must also be provided in the manuscript.

Field-specific reporting

Please select the one below that is the best fit for your research. If you are not sure, read the appropriate sections before making your selection.

[x] Life sciences [ ] Behavioural & social sciences [ ] Ecological, evolutionary & environmental sciences

For a reference copy of the document with all sections, see nature.com/documents/nr-reporting-summary-flat.pdf

Life sciences study design

All studies must disclose on these points even when the disclosure is negative.

Sample size

For the condition optimizing experiments associated with Figure 2:
24 cells associated with the enzyme/protein ratio optimization experiment (3 HeLa cells for each of 8 conditions);
18 cells associated with the LC gradient time optimization experiment (3 HeLa cells for each of 6 conditions);
20 samples associated with the LC-MS repeatability evaluation (10 DDA and 10 DIA).
For the single-cell analysis associated with Figure 3:
256 cells associated with the single-cell analysis experiment (37 A549, 44 HeLa and 27 U2OS cells in DIA mode; 56 A549, 68 HeLa and 24 U2OS cells in DDA mode).
For the quantitative accuracy and precision evaluation associated with Figure 4:
30 samples associated with the quantitative accuracy and precision evaluation of QC (6 samples for each of 5 conditions);
8 samples associated with the quantitative accuracy and precision evaluation of single-cell QC doped with two species (4 samples for each of 2 conditions);
24 samples associated with the quantitative accuracy and precision evaluation of multi-cell samples (4 samples for each of 6 conditions).
For the scratch assay associated with Figure 5:
89 cells associated with the scratch assay (46 migrated HeLa cells; 43 control HeLa cells).

Sample sizes were chosen to include 8-30 single-cell samples for technical experiments to support the validity of the results, and tens to hundreds of single-cell samples for single-cell analyses to give us greater statistical power to identify biological differences.
In general, no calculations were done to determine sample size. Sample size of cellular experiments was determined based on standards for experimental cell biology, attempting to have a minimum of n = 3 biological replicates with sufficient reproducibility. All attempts at data replication were successful.

Data exclusions

No exclusion criteria were pre-established. No data were excluded from the analysis.

Replication

For the condition optimizing experiments associated with Figure 2:

The enzyme/protein ratio optimization experiment (3 biological replications for 8 conditions);
The LC gradient time optimization experiment (3 biological replications for 6 conditions);
The LC-MS repeatability evaluation (10 technical replications in DDA and DIA modes).

For the single-cell analysis associated with Figure 3:
The single-cell analysis experiment (37, 44 and 27 biological replications for A549, HeLa and U2OS cells in DIA mode; 56, 68 and 24 biological replications for A549, HeLa and U2OS cells in DDA mode).

For the quantitative accuracy and precision evaluation associated with Figure 4:
The quantitative accuracy and precision evaluation of QC (6 replications for 5 conditions);
The quantitative accuracy and precision evaluation of single-cell QC doped with two species (4 replications for 2 conditions);
The quantitative accuracy and precision evaluation of multi-cell samples (4 replications for 6 conditions).

For the scratch assay associated with Figure 5:
The scratch assay (46 biological replications for migrated HeLa cells; 43 biological replications for control HeLa cells).

All experimental findings were replicated at least 3 times with enough reproducibility. All attempts at data replication were successful.

Randomization	During the parameter scouting and performance evaluating experiments, the cells were captured randomly and allocated with a random order, and the processing was performed simultaneously and in parallel for all conditions within each experiment. All data collection was done in random order. During the scratch assay experiments, the cells that migrated furthest were assigned to the migrated cell group, while the cells in unmigrated region were randomly captured and assigned to the control group.
Blinding	This is not relevant to our study, because the data was analyzed using unbiased statistics and no covariates exist that could be analyzed with a certain bias.

Reporting for specific materials, systems and methods

We require information from authors about some types of materials, experimental systems and methods used in many studies. Here, indicate whether each material, system or method listed is relevant to your study. If you are not sure if a list item applies to your research, read the appropriate section before selecting a response.

Materials & experimental systems

n/a	Involved in the study	
x		Antibodies
	x	Eukaryotic cell lines
x		Palaeontology and archaeology
x		Animals and other organisms
x		Clinical data
x		Dual use research of concern
x		Plants

Methods

n/a	Involved in the study	
x		ChIP-seq
x		Flow cytometry
x		MRI-based neuroimaging

Eukaryotic cell lines

Policy information about cell lines and Sex and Gender in Research

Cell line source(s)	HeLa (SCSP-504), A549 (SCSP-503) and U2OS (SCSP-5030) cells were purchased from the Cell Bank of the Chinese Academy of Sciences.
Authentication	All cell lines were authenticated by STR profiling.
Mycoplasma contamination	No positive sign of mycoplasma contamination for all cell lines.
Commonly misidentified lines (See ICLAC register)	No commonly misidentified lines were used.

Plants

Seed stocks	*Report on the source of all seed stocks or other plant material used. If applicable, state the seed stock centre and catalogue number. If plant specimens were collected from the field, describe the collection location, date and sampling procedures.*
Novel plant genotypes	*Describe the methods by which all novel plant genotypes were produced. This includes those generated by transgenic approaches, gene editing, chemical/radiation-based mutagenesis and hybridization. For transgenic lines, describe the transformation method, the number of independent lines analyzed and the generation upon which experiments were performed. For gene-edited lines, describe the editor used, the endogenous sequence targeted for editing, the targeting guide RNA sequence (if applicable) and how the editor was applied.*
Authentication	*Describe any authentication procedures for each seed stock used or novel genotype generated. Describe any experiments used to assess the effect of a mutation and, where applicable, how potential secondary effects (e.g. second site T-DNA insertions, mosiacism, off-target gene editing) were examined.*
